# A simple chemical reduction approach to dope β-FeSi_2_ with boron and its comprehensive characterization

**DOI:** 10.1039/d3ra00497j

**Published:** 2023-04-25

**Authors:** Sabyasachi Sen, Debdipto Acharya, Prasanta Kumar Guha, Pallab Banerji, Panchanan Pramanik

**Affiliations:** a Department of Microelectronics & VLSI Technology, Maulana Abul Kalam Azad University of Technology West Bengal 741249 India; b Department of Materials Science, University of Milano-Bicocca Via R. Cozzi 55 Milano I-20125 Italy; c Department of Electronics and Electrical Communication Engineering, Indian Institute of Technology Kharagpur Kharagpur 721302 India; d Material Science Centre, Indian Institute of Technology Kharagpur Kharagpur 721302 India; e Department of Chemistry and Nanotechnology, GLA University Mathura 281406 India pramanik1946@gmail.com

## Abstract

β-FeSi_2_ has been doped with Boron *via* a novel and cost-effective chemical reduction of the glassy phase of [(Fe_2_O_3_ + 4SiO_2_ + B_2_O_3_ + FeBO_3_ + Fe_2_SiO_4_)] using Mg metal at 800 °C. Doped β-FeSi_2_ has been investigated *via* extensive characterization and detailed analysis using first-principles calculations. The reduction in the *d*-spacing as can be observed from the XRD peak shift as well as the blue shift of the β-Raman line along with the right shift of Si and Fe 2p peaks indicate the B doping. The Hall investigation basically demonstrates p-type conductivity. Hall parameters were also analyzed using thermal mobility and dual-band model. The temperature profile of R_H_ demonstrates the contribution of shallow acceptor levels at low temperatures, whereas the deep acceptor level contributes at high temperatures. Dual-band investigation reveals a substantial increase in the Hall concentration with B doping due to the cumulative contribution of both deep and shallow acceptor levels. The low-temperature mobility profile exhibits phonon and ionized impurity scattering just above and below 75 K, respectively. Moreover, it demonstrates that holes in low-doped samples can be transported more easily than at higher B doping. From density functional theory (DFT) calculations, the origin of the dual-band model has been validated from the electronic structure of β-FeSi_2_. Further, the effects of Si and Fe vacancies and B doping on the electronic structure of β-FeSi_2_ have also been demonstrated. The charge transfer to the system due to B doping has indicated that an increase in doping leads to higher p-type characteristics.

## Abstract 2

A simple chemical reduction approach to doping β-FeSi_2_ with boron and its comprehensive characterization through experimental and density functional theorem (DFT) Analyses.

## Graphical abstract

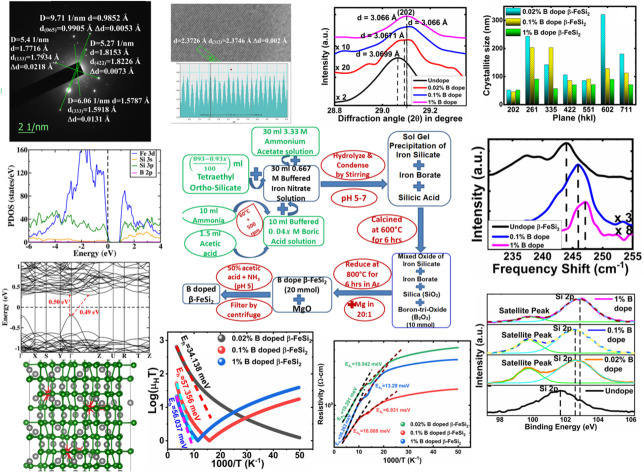

## 1. Introduction

The β-phase of FeSi_2_ has been attracting attention for many years due to its noteworthy semiconducting properties. It is one of those eligible candidates that have the paramount potential for Si-based photovoltaic and optoelectronic applications. It has a direct band gap of 0.85 eV with a wide range of absorption from the infrared to the visible region,^1,2^ a higher optical absorption coefficient (>10^5^ cm^−1^ at 1 eV) compared to that of silicon (∼10^2^ cm^−1^ at 1 eV),^2,3^ moderate hole (*L*_h_) and electron diffusion lengths (*L*_e_) (*L*_e_ ∼ 20 μm at the hole concentration (*p*) of 5 × 10^18^ cm^−3^ and *L*_h_ of 16–38 μm)^4,5^ and the best possibility to be grown epitaxially on a Si substrate with a slight lattice mismatch.^6,7^ In addition, the theoretically simulated conversion efficiency is quite high for β-FeSi_2_-based solar cells (about 16–23% for the β-FeSi_2_/Si hetero-junction solar cell).^8,9^ Thus, it can be a promising and efficient candidate for renewable energy applications. β-FeSi_2_-based solar cells can, therefore, be easily realized on Si substrates because of minimal lattice mismatch (Δ*a* ∼ 2–5.5%).^2,6,7,10–13^ Thus, significant efficiencies of 3.7% and 5.1% have been achieved for hetero-junction and Schottky solar cell structures of n-β-FeSi_2_/p-Si and α-FeSi(Al)/p^+^-Si/n-Si, respectively.^10,11^ Consequently, doping of iron silicide and its detailed physical and electrical analysis through experiments along with its investigation through first principle calculation are very much necessary to comprehend the design and fabrication of β-FeSi_2_-based p–n junction solar cells.

**Fig. 1:**
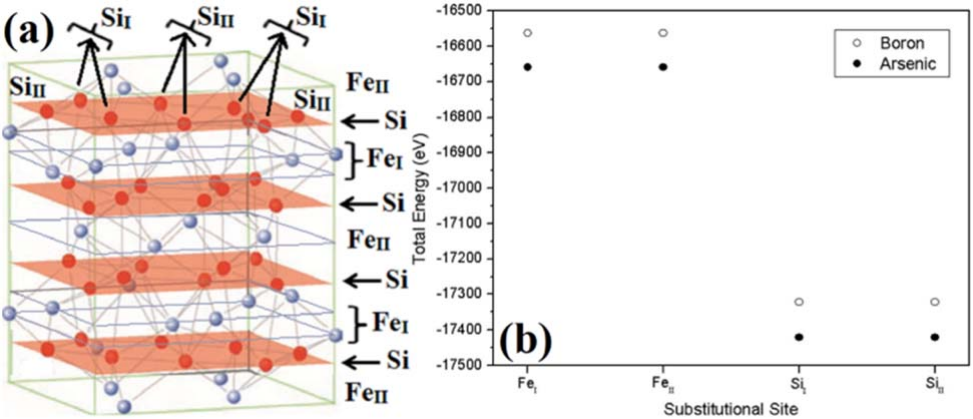
(a) The unit cell of β-FeSi_2_. The light purple spheres represent Fe atoms and the red spheres represent Si atoms. (b) Total energy comparison of As and B-doped β-FeSi_2_ crystals, where As and B atoms substitute different crystallographic sites of Si_I_ and Si_II_ respectively.^16^

Therefore, doping of β-FeSi_2_ by metalloid boron (B) has been investigated in this study. Typically, β-FeSi_2_ has an orthorhombic lattice structure with an unit cell containing 16 formula units distributed over two crystallographically inequivalent sites, 8 Fe_I_, 8 Fe_II_ and 16 Si_I_, 16 Si_II_, as shown in Fig. 1(a).^14,15^ Usually, metalloids like B and As atoms retain lower total energies while replacing Si atomic sites as compared to substituting Fe sites, thus making Si relatively stable sites for B and As doping, whereas Fe sites (Fe_I_ and Fe_II_) are stable sites for transition metal dopants as demonstrated in Fig. 1(b). Furthermore, the As atom prefers a stable Si_I_ site with lower total energies, whereas the B atom generally prefers the Si_II_ site as it retains lower energies in the Si_II_ site, which is indicated in Table 1.^16^ Besides, for the high doping of metalloids like As and B, β-FeSi_2_ lattice will experience sufficient distortion to modulate the Coulomb force acting on Si and Fe atoms to further minimize the overall energy, whereas moderate As and B doping leads to local deformation within the β-FeSi_2_ lattice due to primary interactions between dopant atoms and host Si and Fe atoms.^16^ The relaxation results reveal that the doping of relatively smaller B atoms (*R*_B_ = 0.87 Å and *R*_Si_ = 1.11 Å) leads to the contraction of B–Si and B–Fe bonds, resulting in compressive strain within the β-FeSi_2_ lattice.^16^ On the contrary, doping of the relatively bigger As atom (*R*_Si_ = 1.11 Å and *R*_As_ = 1.15 Å) leads to the elongation of As–Si bonds, resulting in tensile strain within the β-FeSi_2_ lattice.^16^

**Table 1:** Energy differences (Δ*E*) of B and As atoms while substituting different crystallographic sites of Si_I_ and Si_II_ in the β-FeSi_2_ lattice^14,16^

Impurity Element	Substitutional site	Total energy (eV)	Δ*E* (meV)	Preferential substitutional site
B	Si_I_	−17 322.0658	104.20	Si_II_
	Si_II_	−17 322.1700		
As	Si_I_	−17 420.3770	75.90	Si_I_
	Si_II_	−17 420.3011		

Essentially, B doping leads to p-type conductivity in β-FeSi_2_ since there is a lack of one electron per trivalent B atom while forming bonds with adjacent Si and Fe atoms. Thus, the electron deficiency can be complemented by accepting a valence electron from the valence band and thereby leaving behind a hole in the valence band.^16,17^ Carrier concentrations in the range of 10^17^–10^19^ cm^−3^ and mobilities of the order of 20–100 cm^2^ V^−1^ s^−1^ have been reported for B doping.^16,17^ Usually, B doping is reported to introduce shallow impurity levels in the forbidden gap of β-FeSi_2_.^16^ Besides, Arushanov and Tani *et al.* have both proposed dual-band models for doped β-FeSi_2_.^18–20^ However, to the best of our knowledge, there has hardly been any report, so far, concerning detailed analyses on the temperature and doping dependence of β-Fe(Si_1−*x*_B_*x*_)_2_ Hall parameters using thermal mobility and dual-band models. Thus, a study concerning the comprehensive electrical analysis of doped β-FeSi_2_ from the view point of mobility and the dual-band model is essential to develop β-FeSi_2_-based photovoltaic devices.

There have been several reports of doping β-FeSi_2_ with transition metals like Co, Ni, Mn, and Cr within their solubility limits.^18,19,21,22^ However, so far, there have been few reports on doping with metalloids like B and As to alter its semiconducting properties.^16,17^ Most of the doping techniques of β-FeSi_2_ reported so far are based on physical methods. For instance, β-FeSi_2_ has been doped with As and B by the co-sputtering of heavily As-doped Si chip and elemental boron chip targets.^16,17^ Boron and arsenic have also been doped by B^+^ and As^+^ ion implantation at 40 keV on β-FeSi_2_ film deposited on Si substrate.^23^ However, those physical doping techniques often require high-end expensive equipments. On the contrary, the chemical method of doping has the advantages of uniformity and simplicity, and also it does not involve high-end equipment, therefore, it is cost-effective.

Thus, in this report we have proposed a simple and novel chemical reduction technique to dope β-FeSi_2_ with B to make it p-type. This was accomplished by using boric acid (H_3_BO_3_) as the precursor, through the process of synthesizing β-FeSi_2_ nano-particles chemically. Although the diffusion length of minority carriers in β-FeSi_2_ is quite significant, it is still less than that of Si and other semiconducting materials. Therefore, the thickness of the β-FeSi_2_ emitter layer in the solar cell should be a few tens to hundreds of nanometers to attain maximum efficiency.^2,10–13^ Thus, B-doped iron silicide particles should be synthesized in nano-sized order so that they can be further used to fabricate thin nano-metric emitter layers in β-FeSi_2_-based solar cells. The physical and electrical properties of doped materials were comprehensively characterized, as outlined in the following sections of this article. Furthermore, this editorial designates detailed analytical studies of the effects of vacancy defects and B doping on the electronic properties of β-FeSi_2_ by means of first-principles calculation through the *ab initio* density functional theorem (DFT) to validate the experimental studies.

## 2. Experimental & computational details

### 2.1 Experimental procedure

**Fig. 2:**
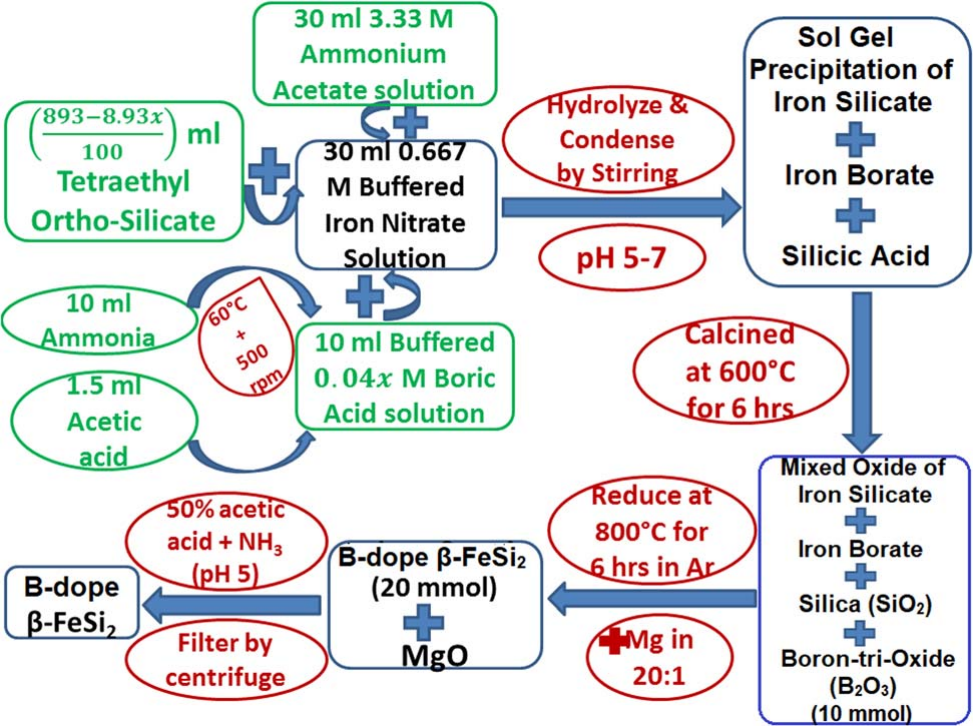
Flow chart of B doping in β-FeSi_2_ using the chemical reduction technique.

Boron-doping of β-FeSi_2_ was carried out by incorporating boric acid (H_3_BO_3_) as the B precursor during sol–gel polymerization in the course of β-FeSi_2_ synthesis as demonstrated in Fig. 2. Since B atom substitutes Si_II_ site, therefore, *x* has been considered as the atomic wt% of B with respect to Si. Initially, 0.667 M of iron nitrate solution buffered with ammonium acetate was added to 4.48 M of (893 − 8.93*x*)/100 ml tetraethyl *ortho*-silicate to prepare a mixed solution. Thereafter, 0.04*x* M of H_3_BO_3_ solution was prepared by dissolving it in 10 ml of ammonia with subsequent heating and stirring, which was made buffered (pH 4–5) with 1–1.5 ml of acetic acid to prevent its precipitation during gel formation. Thus, 0.04*x* M of B^3+^ (in the form of H_3_BO_3_) ammonical solution was successively added to the mixed solution to obtain the desired molar ratio of Fe^3+^ : Si(OC_2_H_5_)_4_ : B^3+^ : NH_3_ = 1 : 2 − 2*x* : 2*x* : 5. Finally, the entire mixed solution was stirred for almost 12 h to obtain homogeneous formation of B^3+^–silicate gel. Subsequently, the gel was calcined at 600 °C for 5–6 h. During calcination, boric acid transformed into boron-tri-oxide by several intermediate steps. Initially, the dehydration of boric acid at 170 °C transformed it into meta-boric acid (HBO_2_), which was further dehydrated to tetra-boric acid [H_2_B_4_O_7_] above 300 °C and thereafter finally it was transformed into boron tri-oxide (B_2_O_3_) at 500 °C.^24,25^ Moreover, calcination at high temperatures also resulted in a complex mixture of ferric silicates and ferric borate (FeBO_3_). Thus, after calcination, the final product of amorphous complex mixtures was reduced by Mg at 800 °C in an Ar atmosphere where relatively smaller B atoms substituted the Si_II_ atomic sites, due to higher kinetic energy, to dope β-FeSi_2_. Thus, the final product of B-doped β-FeSi_2_ consisted of MgO as the by-product of the reduction process. Subsequently, pure B-doped iron silicide was retrieved from the mechanical mixture by washing and centrifuging with 0.5 M of ammonical acetic acid and thereby successive washing with acetone followed by DI water. Finally, three B-doped samples (0.02%, 0.1% and 1%) were prepared where 0.1% B-doped sample was synthesized as nano-particles by just adding double the weight of MgO with Mg metal powder to prevent the agglomeration of silicide during high-temperature reduction.

HRTEM and XRD analyses were performed using JEM-2100 and XPERT PANalytical X-ray diffractometer (with Cu-Kα target), respectively, to investigate the change in lattice inter-planar distances (*d*) to further investigate the B doping. Raman spectroscopy was accomplished using a Horiba Scientific T64000 instrument having an Ar–Kr ion gas laser to investigate the lattice defect line D and shift in β-Raman peaks. XPS was carried out to determine and henceforth to analyze the compositional elements as well as the atomic percentage of dopants. Hall measurement was carried out at room temperature (*T* = 300 K) using Van der Pauw's technique on thin pellets of doped and undoped powder samples of 10 mm diameter by applying 0.545 Tesla magnetic field and 10–20 mA of probe current. The temperature dependence of resistivity of both doped and undoped β-FeSi_2_ had also been investigated within the temperature range of 14–300 K at probe currents of I = 1–10 mA for B doped samples and at I = 50 mA current for undoped β-FeSi_2_. The measurement has been achieved using the four-probe technique.

### 2.2 Theoretical and computational details

The *ab initio* density functional theory calculations have been performed using the Quantum ESPRESSO package to study the electronic structure of undoped and B-doped β-FeSi_2_.^26–28^ A generalized gradient approximation of the PBE form was used to describe the exchange–correlation interaction.^29^ A plane wave basis set was used with wavefunction and charge density cut-offs of 35 Ry and 350 Ry respectively. Interactions between ionic cores and valence electrons were described using ultra-soft pseudopotential.^30^ The Brillouin zone was sampled with a Monkhorst–Pack^31^ mesh consisting of 6 × 6 × 6 *k*-points for the primitive cell of β-FeSi_2_ involving 48 atoms and proportionately equivalent meshes for larger supercells. For B doping, we considered a 2 × 2 × 1 supercell so that the doped concentration was close to that of the experimental concentration. Atomic coordinates were relaxed using the Broyden Fletcher Goldfarb Shanno (BFGS) scheme until the Hellmann–Feynman forces on all atoms were less than 0.001 Ry bohr^−1^.^32–35^ Marzari–Vanderbilt cold smearing with a width of 0.001 Ry was used to achieve convergence.^36^

## 3. Results & discussion

### 3.1 Experimental results

#### 3.1.1 Physical doping analysis

##### 3.1.1.1 Doping analysis by HRTEM

**Fig. 3:**
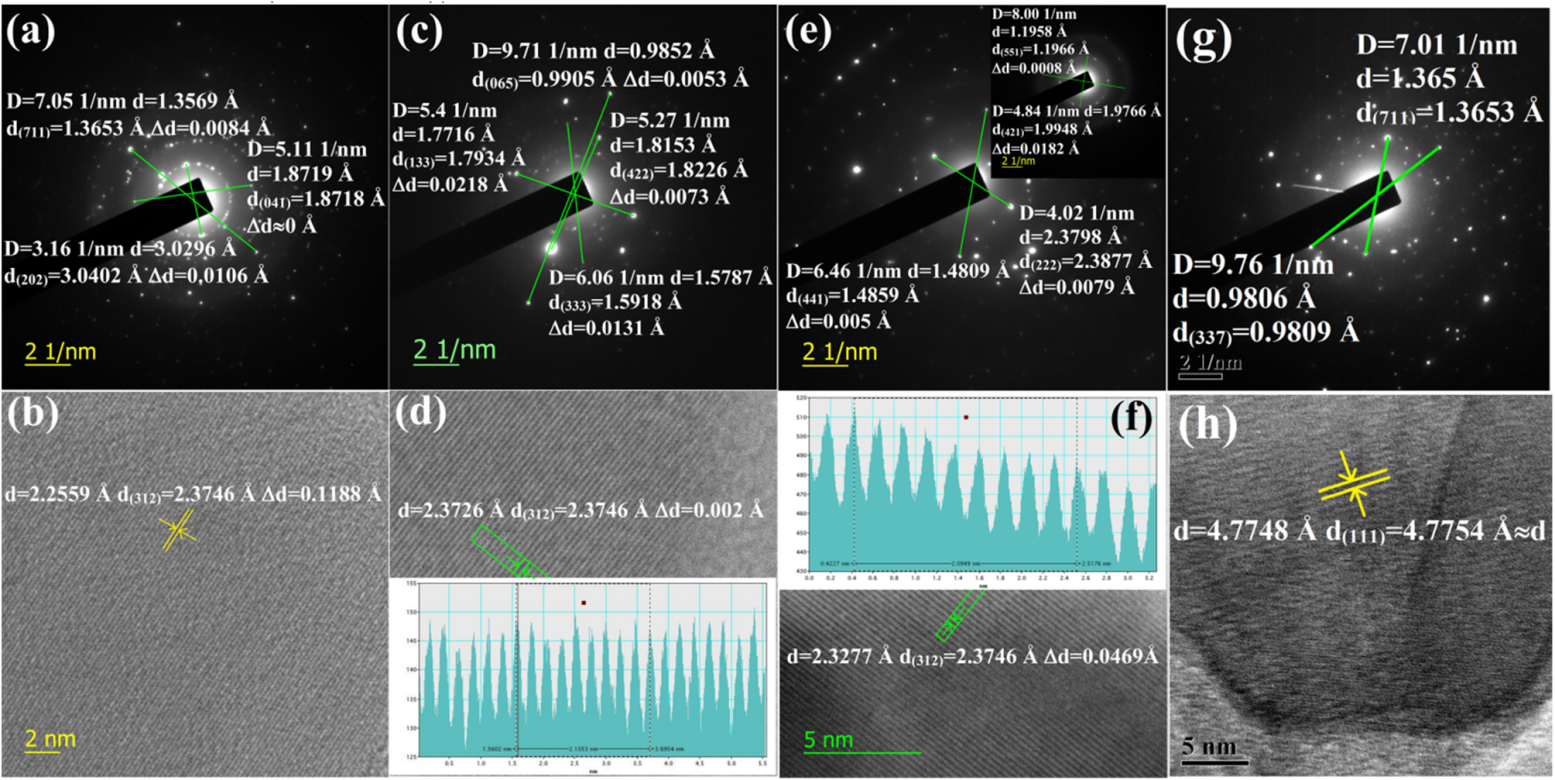
(a) Electron diffraction image of 0.02% B-doped β-FeSi_2_ for (711), (041) and (202) planes; (b) The lattice image of 0.02% B-doped β-FeSi_2_ for (312) plane; (c) Electron diffraction image of 0.1% B-doped β-FeSi_2_ for (065), (133), (422) and (333) planes; (d) Lattice image of 0.1% B-doped β-FeSi_2_ for (312) plane; (e) Electron diffraction image of 1% B-doped β-FeSi_2_ for (222) and (441) planes and in the inset for (551) and (421) planes; (f) Lattice image of 1% B-doped β-FeSi_2_ (verified from JCPDS data card 04-007-1080 and 01-079-5663); (g) Electron diffraction image of undoped β-FeSi_2_ for (711) and (337) planes and (h) Lattice image of undoped β-FeSi_2_.

The doping of numerous samples, *i.e.*, Li^+^-doped Si nano-wires and Zn-doped SnO_2_, has been studied by lattice and electron diffraction profiles, which has motivated us to investigate β-FeSi_2_ doping with the help of electron diffraction studies.^37,38^ The electron diffraction profile in Fig. 3(g) reveals that there is almost no changes in the *d* spacing of (711) and (337) planes of undoped β-FeSi_2_. On the contrary, the electron diffraction profiles of B-doped β-FeSi_2_ in Fig. 3(a), (c) and (e) demonstrates a significant reduction in *d* spacing within the range of 0.0008–0.02 Å depending on both the planar orientation and doping percentage as summarized in Table 2. This is in good agreement with the effects of B doping on the β-FeSi_2_ lattice, revealing the contraction of major bonds like B–Si (B–Si_I_ by −4.844% and B–Si_II_ by −5.489%) and B–Fe (B–Fe_I_ by −4.846% and B–Fe_II_ by −6.741%) bonds. This contraction of bonds length leads to compressive strain, introducing a reduction in inter-planar spacing (*d*).^16^ The profiles in Fig. 3(a) and (c) reveal that the *d* spacing decreases significantly for (711) and (202) planes of 0.02% B-doped samples as well as for the (065), (422), (133) and (333) planes of 0.1% B-doped samples, whereas there is no change at all for the (041) plane of 0.02% B-doped samples. Thus it substantiates that B atoms are not uniformly incorporated into all the lattice planes and therefore indicates that doping is preferred by some specified planes. Consequently, more doping occurs in some favourable planes and there is less in other planes.

**Table 2:** Summary of changes (Δ*d*) in the interplanar spacing of significant planes for various B dopings

Decrease in interplanar spacing	B-doped β-FeSi_2_			Decrease in interplanar spacing	B-doped β-FeSi_2_		
	0.02%	0.1%	1%		0.02%	0.1%	1%
Δ*d*_(202)_ (Å)	0.0106	—	—	Δ*d*_(222)_ (Å)	—	—	0.0079
Δ*d*_(041)_ (Å)	0	—	—	Δ*d*_(441)_ (Å)	—	—	0.005
Δ*d*_(711)_ (Å)	0.0084	—	—	Δ*d*_(551)_ (Å)	—	—	0.0008
Δ*d*_(065)_ (Å)	—	0.0053	—	Δ*d*_(421)_ (Å)	—	—	0.0182
Δ*d*_(422)_ (Å)	—	0.0073	—	Δ*d*_(023)_ (Å)	—	—	—
Δ*d*_(133)_ (Å)	—	0.0218	—	Δ*d*_(224)_ (Å)	—	—	—
Δ*d*_(333)_ (Å)	—	0.0131	—	Δ*d*_(312)_ (Å)	0.1188	0.002	0.0469

The electron diffraction profile of 1% B doped sample in Fig. 3(e) also reveals a significant reduction in *d* spacing. Besides, the lattice investigation also agrees with the results of the diffraction profile that the reduction in *d* spacing varies from plane to plane for a specific doping percentage as shown in Fig. 3(b), (d), (f) and (h). Conversely, for a specific lattice (312) plane, the *d* spacing decreases, *i.e.*, Δ*d* increases quite significantly from 0.002 to 0.047 Å as the B doping increases from 0.1 to 1%. This is probably due to the incorporation of an increased number of smaller B atoms into larger Si atomic sites. Also, Δ*d* of the (421) plane for 1% B doping agrees quite well with the electron diffraction profile. Since the B atomic radius (*R*_B_ = 0.87 Å) is almost 0.24 Å smaller than that of the Si atomic radius, therefore, the incorporation of B dopant will lead to the modulation of the interplanar spacing by at least 0.1–0.2 Å, which is reflected in Fig. 3(f). Thus, a significant reduction in the interplanar spacing (Δ*d*) for several lattice planes indicates the fact of B doping.

##### 3.1.1.2 Doping analysis by X-ray diffraction

**Fig. 4:**
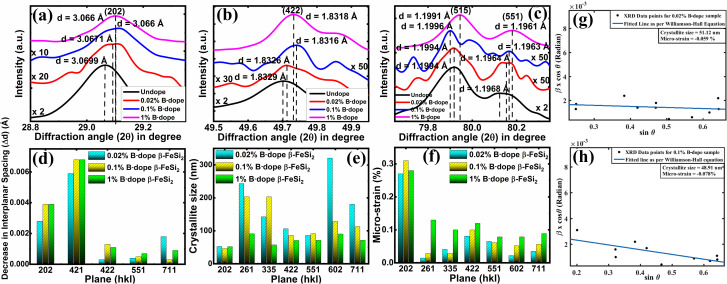
XRD peak shift of the (a) (202), (b) (422), and (c) (515) and (551) planes of B-doped β-FeSi_2_ with respect to the undoped one (verified from JCPDS data card 04-007-1080 and 01-079-5663); (d) Decrease (Δ*d*) in the interplanar spacing change as a function of planar orientation for several B-doped β-FeSi_2_; Plots of (e) crystallite size as a function of significant lattice planes for several B-doped β-FeSi_2_ and (f) micro-strain as a function of significant lattice planes for several dopings of B; Fitted straight line according to the Williamson–Hall model to determine crystallite size and compressive strain in (g) 0.02% and (h) 0.1% B-doped β-FeSi_2_.

The impurity doping prompted an indirect change in the lattice structure, which can be well verified by the change in the interplanar spacing (*d*) as already stated. Fig. 4(a)–(c) shows XRD peak shifts of doped β-FeSi_2_ with respect to the undoped one for some significant (202), (422), (515) and (551) planes. Since B atoms substitute the Si_II_ atomic sites, therefore, B doping induces compressive strain between two consecutive parallel planes due to the shrinkage of bond distances, resulting in the gradual reduction of the *d* spacing^14,16^ and consequently, the XRD peaks shift towards higher 2*θ* as B doping increases from 0.02% to 1% as per Bragg's law, which is revealed in Fig. 4(a)–(c); thus, it justifies B doping for certain substantial planes. Similarly, a gradual reduction in *d* spacing with the increase in B doping, due to compressive lattice strain, is also evident from Fig. 4(d). This agrees with the fact that B–Si and B–Fe bond lengths contract with B doping, which further causes compressive strain to result in diminished *d* spacing as discussed earlier.^14,16^ Besides, modulation in interplanar spacing (Δ*d*) also depends on the lattice planes, *i.e.*, varies from plane to plane for a specified doping amount as shown in Fig. 4(d). Therefore, the average modulation in the inter-planar distance (*d*) for some specified planes like (422) is quite significant, of the order of 0.001 Å, whereas the average Δ*d* for other planes like (515) is almost 0.0006 Å, which is quite trivial. It is also very clear from Fig. 4(d) that for a specific B doping, Δ*d* is reduced with an increase in the planar orientaion from (421) to (551).

Moreover, the crystallite size as well as the micro-strain of doped samples have been deployed for certain substantial planes by fitting plotted XRD data with the Williamson–Hall straight line equation as shown in Fig. 4(g) and (h),^39^

1 *β* cos *θ* = 4*ε* sin *θ* + *Kλ*/*L*

where *K* = the Scherrer constant = 0.94, *λ* = 1.5406 Å is the Cu target Kα_1_ X-ray wavelength, *β* is the FWHM in radians, 2*θ* is the diffraction angle, *L* is the crystallite size and *ε* is the lattice micro-strain. From Fig. 4(f) it can be inferred that for a specific lattice plain, as the doping increases, more dopant atoms substitute for Si lattice sites, which further leads to a significant increase in compressive lattice strain. Therefore, when the strain exceeds the critical value, larger crystallites break down into smaller crystallites. As a consequence, the crystallite size becomes smaller and smaller with the increase in doping from 0.02% to 1% for a specified plane [Fig. 4(e)]. Besides, micro-strain is negative for B-doped samples, which indicates the compressive strain. As B doping increases from 0.02% to 0.1% the compressive strain is enhanced from −0.059% to −0.078%, thus, leading to a reduction in crystallite size from 51.12 to 48.91 nm as can be seen from Fig. 4(g) and (h).

##### 3.1.1.3 Doping analysis by Raman spectroscopy

**Fig. 5:**
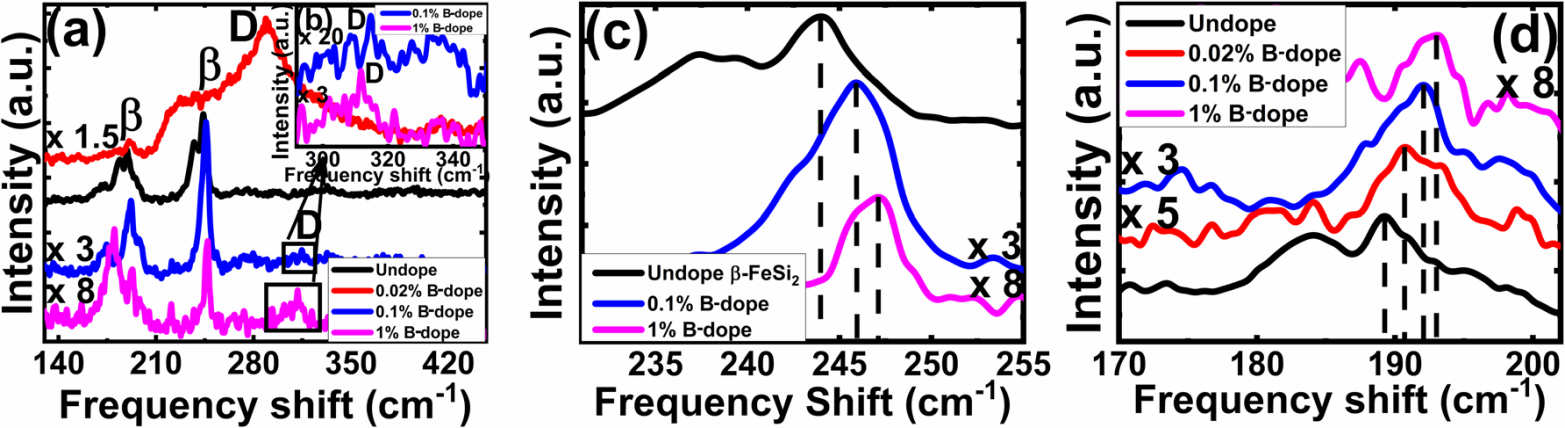
(a) Raman spectroscopy of undoped and several percentages of B-doped β-FeSi_2_; (b) The inset shows magnified D defect peaks for 0.1% and 1% B-doped β-FeSi_2_; Blue shift of the β-peak for several B-doped samples at (c) 189.27 cm^−1^ and (d) 243.96 cm^−1^ frequencies.

The comparative Raman spectra of undoped and doped β-FeSi_2_ in Fig. 5 confirms the fact of doping. The characteristic Raman lines (β) for the undoped sample at 244 and 190 cm^−1^ are resulted from Raman active (Ag) modes due to first-order Raman scattering.^40^ On the other hand, for B-doped β-FeSi_2_, the β-Raman peak is blue-shifted towards higher frequency with an increase in doping as shown in Fig. 5(a), (c) and (d). This is due to the reduction in bond length as the smaller B atom (*R*_B_ = 0.87 Å) forms a shorter co-valent bond with adjacent Fe and Si atoms while substituting for the larger Si_II_ atom (*R*_Si_ = 1.11 Å) as compared to the conventional Si–Si and Si–Fe bonds in undoped β-FeSi_2_.^16^ This is in good agreement with the observations of Tan *et al.*, *i.e.* B–Si_I_, B–Si_II_, B–Fe_I_ and B–Fe_II_ bond lengths diminished by 4.8, 5.5, 4.8 and 6.7%, respectively, due to B doping in β-FeSi_2_.^16^ Thus, the binding energies of those shorter co-valent bonds are quite higher than those of conventional Si–Si and Si–Fe bonds. Therefore, higher energy photons are required to excite and vibrate those stronger bonds, which leads to the blue shifting of β-peaks towards higher frequencies with an increase in B doping [Fig. 5(c) and (d)]. Thus, B doping shortens the atomic bond length, resulting in β-FeSi_2_ lattice distortion, which further minimizes the total energy of the system to make it stable.^14,16^ An additional D peak at 300 cm^−1^ is probably due to defect-induced Raman scattering originating from lattice imperfections and stress introduced by dopants, which is shown in Fig. 5(a) and (b). There is also certain probability of smaller boron atoms occupying interstitial space in the β-FeSi_2_ lattice to cause further lattice imperfections and thus lattice defects. These lattice defects cause compressive strain to promote a defect (D) peak at 300 cm^−1^ as exhibited in Fig. 5(a) and (b). Besides, Fig. 5(a) reveals that the blue-shifted β peak at 244 cm^−1^ is merged with the defect (D) peak at 300 cm^−1^, leading to a broader shoulder around 230 cm^−1^. Subsequently, defect line D is intensified and shifted towards the blue wavelength with an increase in dopant concentration. This is due to a reduction in bond length induced by increased lattice defects.^14,16^ Thus, the blue shift of β and D peaks with an increase in doping designates effective B doping in β-FeSi_2_.

##### 3.1.1.4 Doping analysis by X-ray photoelectron spectroscopy

**Fig. 6:**
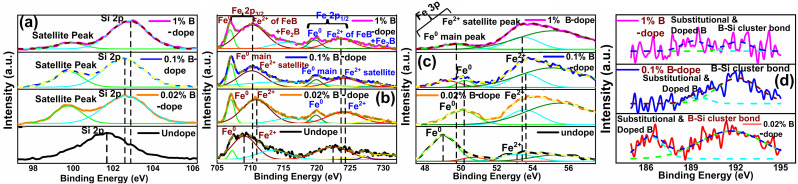
Upshift of (a) Si 2p XPS peak, (b) Fe 2p^3^ XPS peak and (c) Fe 3p XPS peak for several percentages of B-doped β-FeSi_2_ with respect to the undoped one; (d) XPS peak B 1s and Si–B bonding for several B-doped samples.

Fig. 6 demonstrates the XPS spectra for doped as well as undoped β-FeSi_2_. For undoped β-FeSi_2_, the binding energies of Si 2p and Fe 2p^3^ peaks are 100.14 and 707.7 eV, respectively. A significant upshift in the Si 2p binding energy has been perceived in Fig. 6(a) for B-doped samples w.r.t. the undoped one. This is the consequence of the reduction in Si–B and Fe–B bond lengths due to the substitution of the larger Si atom by the smaller B atom.^16^ Consequently, Si–B and Fe–B binding energies become stronger where higher photon energy is necessary to discharge an electron from the stronger bond, leading to an upshift of the Si 2p peak. Besides, the FWHM of the Si 2p peak becomes substantially broader with the increase in B doping due to a predominant increase in structural disorder induced by defects. There is a likelihood for B atoms to be settled at the interstitial sites of the β-FeSi_2_ lattice.^16^ Thus, the up-shift of the Si 2p peak and the broadening of FWHM justify the B doping of β-FeSi_2_.

It can be observed from Fig. 6(b) that the Fe 2p spectrum of doped β-FeSi_2_ is split into two major peaks: (i) Fe 2p_3/2_, which consists of two foremost peaks: (a) main peak of the Fe^0^ state at 707.04 eV and (b) the satellite peak of the Fe^2+^ state at 710.36 eV; (ii) Fe 2p_1/2_, which consists of two major peaks: (a) Fe^0^ main peak at 720.1 eV and (b) Fe^2+^ satellite peak at 723.16 eV. The Fe^2+^ satellite peak mainly forms due to the splitting of the 2p peak owing to the exchange interaction between the antiparallel electron spin of the 2s core and 2p unfilled orbitals, whereas Fe^0^ peaks are due to the state where the 2s core electron spin is parallel to the 2p electron spin.^41,42^ Therefore, it can be observed from Fe 2p^3^ spectrum that there is a noteworthy upshift of the Fe^2+^ states of both Fe 2p_3/2_ and Fe 2p_1/2_ peaks for B-doped samples, which can be attributed to the contraction of the Fe–B bonds, essentially due to B doping.^14,16^ Besides, the FWHM of both Fe 2p_3/2_ and Fe 2p_1/2_ peaks becomes more and more wide mainly due to an increase in defect densities due to the tendency of B atoms to be incorporated within interstitial sites.

Similarly, the Fe 3p peak of the B doped β-FeSi_2_ has been split into two major peaks: (i) a main peak of the Fe^0^ state at 49.86 eV due to the 3p core electron spin parallel to the 3d electron spin and (ii) another satellite peak of the Fe^2+^ state at 53.51 eV due to the 3p spin antiparallel to the 3d spin as has been revealed in Fig. 6(c).^41^ As can be clearly observed from Fig. 6(c), both Fe^0^ and Fe^2+^ peaks are considerably upshifted since higher X-ray energy is required to discharge an electron from the Fe–B co-valent bond, which becomes significantly shorter (Fe_I_–B and Fe_II_–B by −4.8% and −6.7% respectively) and, thereby, stronger after substitution by B atoms.^16^ The FWHM of the Fe 3p peak widens with an increase in B doping as there is an enhanced probability of more B atoms being incorporated interstitially to induce more defects.^41^ Thus an extensive shift of Fe 2p and 3p peaks and the widening of their corresponding FWHM indicate effective doping with B.

There is a distinct B 1s peak at approximately 186–189 eV as revealed in Fig. 6(d), which indicates B doping within β-FeSi_2_.^43^ Besides, Fig. 6(d) clarifies the XPS peak of the B–Si bond within the doped β-FeSi_2_ lattice at 192 eV.^43,44^ As the B doping increases, both the elemental B 1s and Si–B binding energies upshift significantly due to a gradual reduction in the Si–B bond length.^16^ Thus, the significant up-shift of B 1s as well as Si–B binding energy confirm the fact of B doping.

#### 3.1.2 Electrical analysis

##### 3.1.2.1 Doping analysis by the Hall effect

Hall mobility has been perceived for B-doped β-FeSi_2_ by Hall measurements. Carrier mobility was also calculated from the Caughey–Thomas model as follows:^45^2
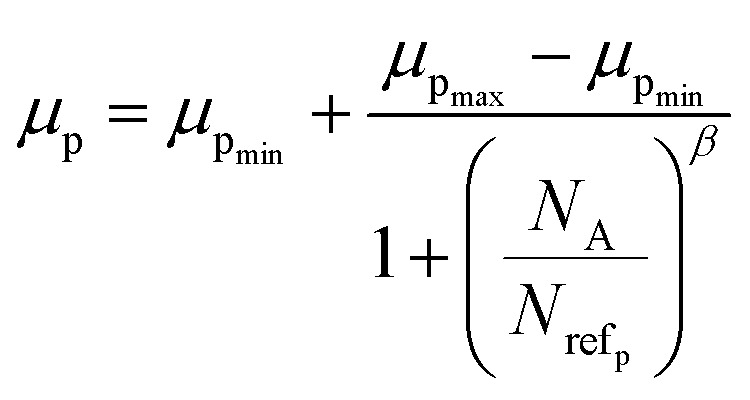
where *μ*_p_ is the majority carrier hole mobility, *μ*_p_min__ is the minimum hole mobility at higher carrier concentration, *μ*_p_max__ is the maximum hole mobility at a lower concentration, *N*_A_ is the acceptor concentration, *N*_ref_p__ is the roll-on concentration and *β* is the fitting parameter. Values of mobility parameters were taken from the reported work of Yuan *et al.* as shown in Table 3.^46^

**Table 3:** Caughey–Thomas mobility model parameters for β-FeSi_2_ (ref. 46)

Materials	Carriers	*μ*_max_ (cm^2^ V^−1^ s^−1^)	*μ*_min_ (cm^2^ V^−1^ s^−1^)	*N*_ref_ (×10^17^ cm^−3^)	*α*/*β*
β-FeSi_2_	Hole	256	0.5	1.2	0.82

The dopant activation energy (*E*_A_) has also been calculated theoretically at room temperature from the dual-band model proposed by Arushanov and Tani *et al.*,^18,20^3
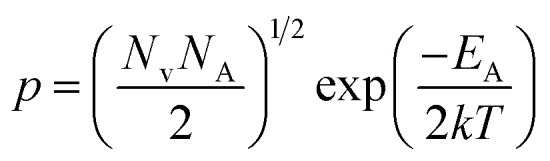
where4
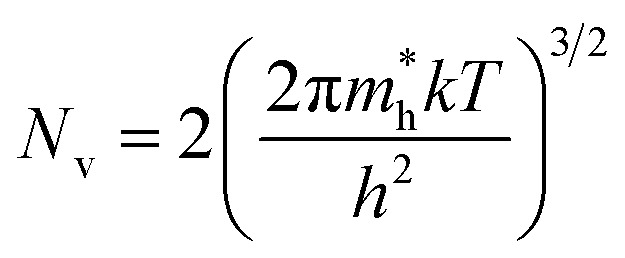
*N*_A_ is the acceptor concentration, *E*_A_ is the acceptor activation energy, 
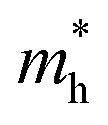
 is the effective mass of the hole and *N*_v_ is the valence band density, which was calculated to be 2.508 × 10^19^ cm^−3^ considering 
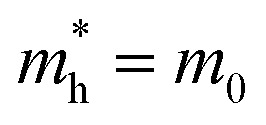
, where *m*_0_ is the rest mass.^47^ Besides, the acceptor concentration (*N*_A_) has also been derived from the dopant atomic wt% using following formulae,

5 *N*_A_ = (*x* × *M* × *N*_Avagadro_)/(*v*_FeSi_2__ × 10^5^)

where *x* is the B atomic percentage, *M* = 40 mmol is the molar mass of boric acid, *N*_Avagadro_ is Avogadro's number, *v*_FeSi_2__ = 0.472 cm^3^ is the volume of 20 mmol β-FeSi_2_ obtained after Mg reduction. The measured Hall parameters along with the calculated acceptor activation energy *E*_A_ and hole mobility are summarized in Table 4.

**Table 4:** Measured Hall parameters and calculated activation energy for B-doped β-FeSi_2_

B Atomic wt%	Hall Co-efficient (m^3^ C^−1^)	Cal. acceptor conc. (cm^−3^)	Hall conc. (cm^−3^)	Activation energy (meV)	Cal. mob. (cm^2^ V^−1^ s^−1^)	Hall mob. (cm^2^ V^−1^ s^−1^)	Resistivity (Ω cm)
0.02%	+1.81 × 10^−1^	10^19^	3.46 × 10^19^	−58.33	2.93	2.18	8.29 × 10^−2^
0.1%	+9.18 × 10^−3^	5 × 10^19^	6.87 × 10^20^	−171.24	1.104	1.75 × 10^−1^	5.24 × 10^−2^
1%	+8.13 × 10^−3^	5 × 10^20^	1.6 × 10^21^	−155.42	0.575	7.21 × 10^−2^	5.42 × 10^−2^
Undoped	+2.74	2.05 × 10^18^	4.38 × 10^18^	7.57	13.21	8.9	3.07 × 10^−1^

The average Hall co-efficient *R*_H_ of B-doped samples is positive, indicating the sample to be p-type, which agrees quite well with the literature.^16,17,23^ On the other hand, it is gradually reduced from +2.74 m^3^ C^−1^ to +8.13 × 10^−3^ m^3^ C^−1^ as β-FeSi_2_ is drifted from undoped to 1% B-doped conditions, thus indicating β-FeSi_2_ to be more and more p-type with the increase in B doping. Here, the negative activation energy (*E*_A_) indicates the existence of dual levels comprised of a shallow acceptor band due to defects and a deep acceptor level due to B dopants.^18,20^ Hall (hole) concentration increases consistently from 4.38 × 10^18^ cm^−3^ to 1.6 × 10^21^ cm^−3^ as the doping increases from undoped to 1% B-doped condition, which is quite expected. Particularly, the Hall concentration is significantly higher than that of the calculated acceptor concentration in doped samples, which can be attributed to the following well-defined justifications: (i) generation of innumerable defect states while performing doping, and hence, (ii) the existence of dual bands/levels in doped β-FeSi_2_ with a deep acceptor level due to B dopant and a shallow acceptor level due to structural defects. This shallow defect level thus contributes additional carriers to the valence band apart from the contribution from dopant level.^18,20^ Therefore, the dual-band model allows the hole concentration (*p*) in the B-doped β-FeSi_2_ to be realized in terms of two acceptor levels^20^6
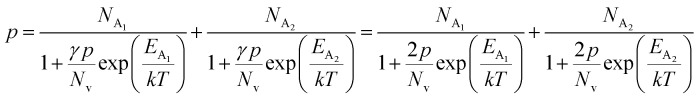
where *γ* = 2 is the degeneracy factor for β-FeSi_2_, *N*_A_1,2__ and *E*_A_1,2__ are the concentration and activation energies of the shallow and deep acceptor levels, respectively and *T* is the lattice temperature. At low temperatures, holes in B-doped β-FeSi_2_ will be activated mainly from the shallow defect level where the deep dopant level will not contribute at all, thereby, causing low hole concentration.^20^ On the contrary, at intermediate, as well as high temperatures, both the deep and shallow acceptor levels play a dynamic role in providing carriers to the valence band essentially due to sufficient thermal energy.^20^ Therefore, at room temperature, as well as at high temperatures, overall hole contribution is much higher than that of hole contribution due to only deep acceptor level. On the contrary, the acceptor concentration is basically calculated by considering only the B atoms in the deep acceptor level and neglecting the contribution of the shallow defect level. Thus, the dual-band model and defect generation due to B doping clarify the background reason for substantially higher Hall concentration than that of the calculated acceptor concentration.

The average resistivity also declines gradually from 3.07 × 10^−1^ Ω cm to 5.42 × 10^−2^ Ω cm with an increase in B doping from undoped to 1% doped conditions. The Hall mobility consistently reduces from 8.9 cm^2^ V^−1^ s^−1^ to 7.21 × 10^−2^ cm^2^ V^−1^ s^−1^ with the gradual increase in B doping mainly due to ionized impurity and carrier–carrier scattering. There is also the likelihood of enhanced scattering by dislocations and stress created by the probable incorporation of B atoms into the interstitial sites, which also causes mobility degradation. Moreover, the hole mobility of doped micro-crystalline β-FeSi_2_ can also be clarified by a small polaron model at 300 K,^48^7
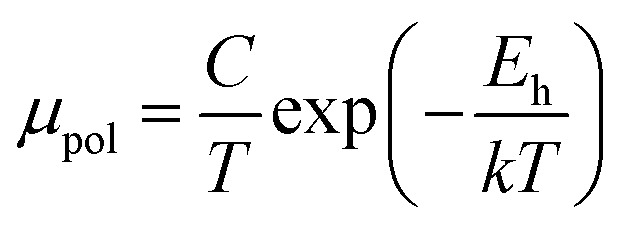
where *E*_h_ is the hopping energy of holes and *C* is a constant independent of the temperature. Thus, the investigated Hall coefficient and other Hall parameters indicate the effective B doping in β-FeSi_2_.

##### 3.1.2.2 Doping analysis from low-temperature resistivity

**Fig. 7:**
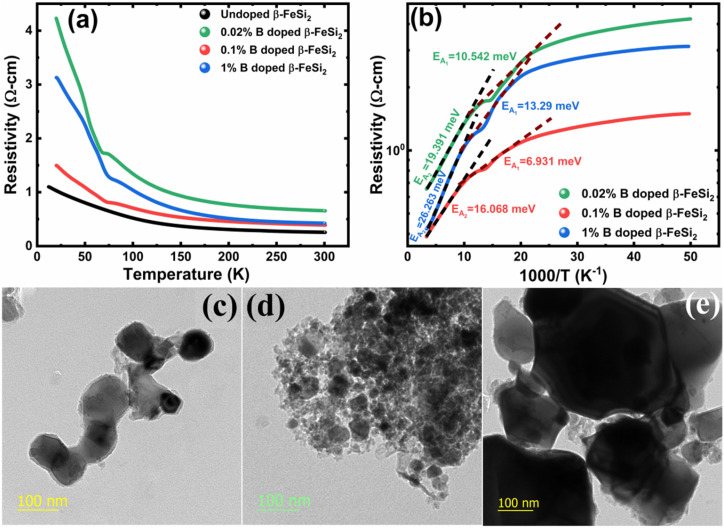
(a) Electrical resistivity of 0.02%, 0.1% and 1% B-doped β-FeSi_2_ w.r.t undoped β-FeSi_2_ as a direct function of temperature; (b) Logarithmic resistivity of 0.02%, 0.1% and 1% B-doped β-FeSi_2_ as a function of reciprocal absolute temperature; HRTEM image of (c) 0.02% B-doped β-FeSi_2_, (d) 0.1% B-doped β-FeSi_2_ and (e) 1% B-doped β-FeSi_2_.

The low-temperature resistivity curve of β-FeSi_2_ for several percentages of B doping is shown in Fig. 7(a). The resistivity (*ρ*) decays exponentially with an increase in temperature, thus, indicating all the doped samples to be semiconducting. Here, the nature of the resistivity curve can be designated by the following justifications: (i) as usual reduction in resistivity with an increase in doping due to an increase in dopant atoms in the acceptor level; (ii) an increase in bandgap due to substantial doping with semiconductor-like B, having a reasonably higher bandgap than that of β-FeSi_2_ and (iii) resistivity validation by the dual-band model.^18,20,49–51^ The resistivity (*ρ*) of B-doped β-FeSi_2_ is considerably higher than that of the undoped one. This is because of the increase in the bandgap from the *E*_g_ value (0.87 eV) of β-FeSi_2_ to the *E*_g_ value (1.6–2.0 eV) of B through the formation of β-Fe(Si_1−*x*_B_*x*_)_2_, as well as defect strain deformation due to B-doping in interstitial lattice sites.^49–51^ Essentially, there is a clear contention between the contributions of the acceptor level and the increase in bandgap due to B doping. The bandgap increase dominates over the contributions of the acceptor level leading to an effective increase in resistivity. Subsequently, with the increase in B doping from 0.02% to 1%, the resistivity (*ρ*) tends to decrease from 0.66 Ω cm to 0.4 Ω cm at room temperature as expected. Only the exceptional fact is the marginal reduction of resistivity of the 0.1% B-doped sample as compared to that of the 1% B-doped β-FeSi_2_ explicitly in the low-temperature zone which agrees quite well with the Hall result in Table 4. This is due to the nano-nature of the 0.1% B-doped β-FeSi_2_ as compared to the 1% B-doped sample as cited in Fig. 7(c)–(e). As already mentioned, the transport properties of B-doped β-FeSi_2_ can be explained by the dual-band model with the existence of a shallow defect level and another deep dopant level.^18,20^ Thus, doped nano-particles have much higher structural defects in nano-grain boundaries as compared to their micro-grain counterparts. Therefore, nano β-FeSi_2_ will form numerous defect levels to form broader shallow acceptor band, which lies in close proximity to the valence band as compared to the narrow defect band in doped micro-particles. Consequently, doped β-FeSi_2_ nano-particles provide a larger number of holes to the valence band, resulting in substantially lower resistivity than the micro-particle counterpart. Thus, it is pretty clear that both B doping and particle size play vital roles in determining the resistivity of doped β-FeSi_2_.

Hole (Hall) concentration (*p*) in B-doped β-FeSi_2_ can be realized in terms of two acceptor levels as per the dual-band model as mentioned earlier in eqn (6).^20^ The first term 
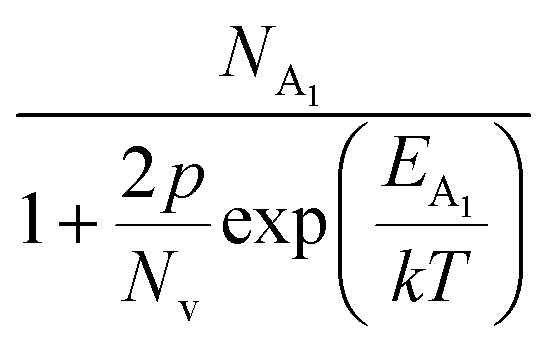
 is the contribution due to the shallow defect band with activation energy *E*_A_1__ and the second term 
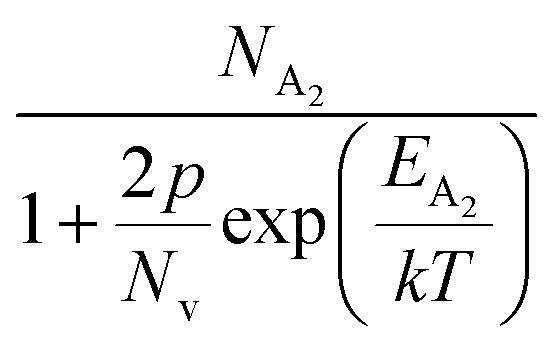
 is the contribution due to a deep acceptor level with energy *E*_A_2__. The shallow defect band contributes at low temperatures, within 50–150 K, whereas the deep acceptor level contributes at high temperatures, above 100–150 K. Therefore, at low temperatures, neglecting the effects of the deep acceptor level and considering the ionization of the shallow acceptor level, the hole concentration (*p*) can be given as follows:^20,52^8
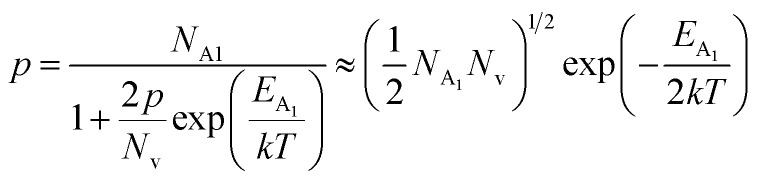


At high temperatures, disregarding the effects of the shallow defect band and considering 
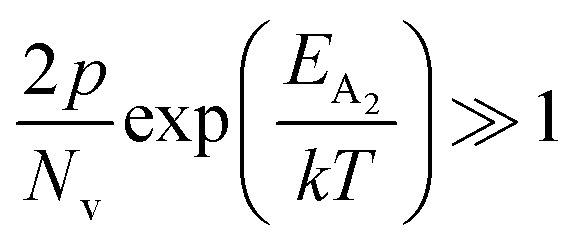
, the hole concentration (*p*) in eqn (6) for doped β-FeSi_2_ can be expressed as follows:^20^9
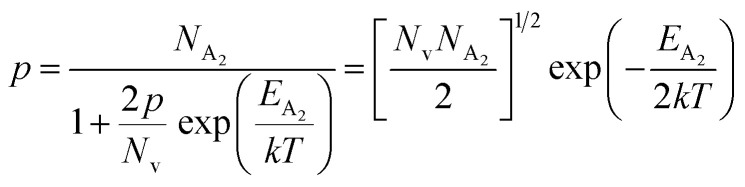


Therefore, applying resistivity 
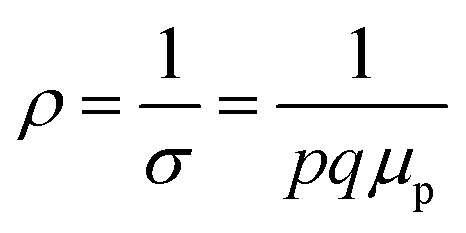
eqn (8) and (9) can be revised as follows:10
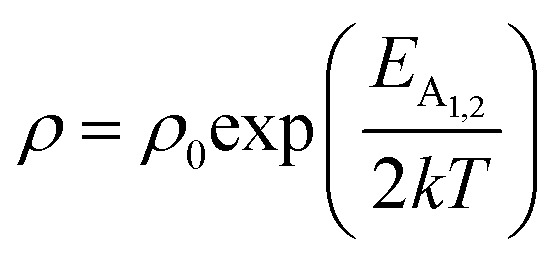
where 
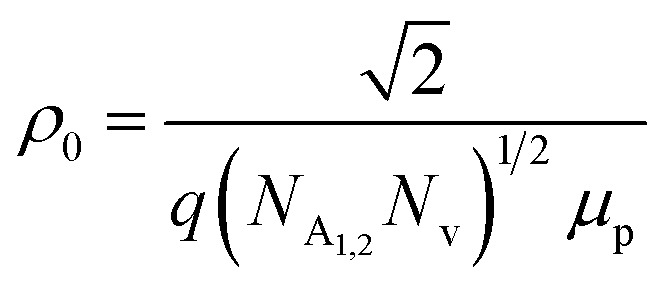
 is a proportionality constant, q is the electronic charge, *σ* is the conductivity and *μ*_p_ is the mobility of the holes. Therefore, taking log on both sides, eqn (10) can be reviewed as follows:11
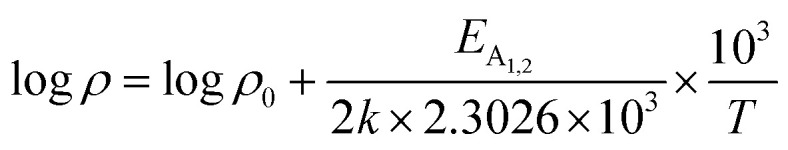


Thus, eqn (11) portrays a linear relationship between log *ρ* and the inverse of temperature (1/*T*) in the high-temperature regime, which is revealed in Fig. 7(b) for different percentages of B doping. Therefore, the activation energies of the shallow acceptor band (*E*_A_1__) and the deep acceptor level (*E*_A_2__) can be determined from the gradients of straight lines at the high (100–300 K) and low temperature (50–100 K) regimes in Fig. 7(b). The shallow defect band activation energies of 0.02% and 1% B-doped β-FeSi_2_ are 10.542 meV and 13.29 meV, respectively whereas deep acceptor level energies of 0.02% and 1% B-doped β-FeSi_2_ are 19.391 meV and 26.263 meV, respectively. On the other hand, the activation energy of the shallow defect band (*E*_A_1__ = 6.931 meV) of the 0.1% B-doped sample is considerably lower than that of the 0.02% and 1% B-doped β-FeSi_2_, mainly due to larger defect densities in the 0.1% doped sample because of its nanoparticle size. Similarly, the activation energy of the deep acceptor level (*E*_A_2__ = 16.068 meV) of the 0.1% B-doped sample also becomes remarkably lower than that of the 0.02% B-doped β-FeSi_2_, essentially due to the higher doping of the 0.1% B-doped sample.

##### 3.1.2.3 Analysis of Hall parameters from the thermal component of mobility

The temperature dependence of Hall parameters can also be precisely derived from the thermal component of mobility. Carrier mobility (*μ*_H_) in the doped semiconductor depends on the temperature in two different ways: (i) mobility at higher temperatures is inversely proportional to *T*^*n*^ (*μ*_L_ ∝ *T*^−*n*^), where *n* varies from 1.8 to 2.1.^53^ Therefore, mobility at high temperatures cannot solely be elucidated by acoustic-phonon scattering (*n* = 1.5), rather, it could be justified by polar-optical-phonon, inter-valley, ionized impurity and grain boundary scatterings also.^53^ (ii) Secondly, mobility at lower and medium temperatures due to ionized impurity scattering is directly proportional to *T*^3/2^ and inversely proportional to the ionized impurity concentration (*N*_I_) 
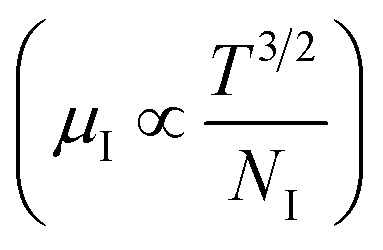
 since the probability of the scattering effect decreases with an increase in temperature due to an increase in random thermal velocity which further minimizes the coulombic interactions between carriers and ionized impurity centers.^54^ Most of our doped samples are sub-micron in nature and polar-optical-phonon and grain boundary scatterings are a quite complex phenomenon, as well as some of their complex parameters like high-frequency dielectric constant (*ε*_∞_), Debye temperature (*θ*) and effective barrier height at grain boundaries (*ϕ*_eff_) are quite difficult to experimentally characterize; therefore, the two simplest forms of scatterings, *i.e.* acoustic-phonon scattering and ionized impurity scattering have been considered to analyze our mobility model.^53,54^ Therefore, mobility due to acoustic-phonon and ionized impurity scattering can be respectively given as follows:^53^12
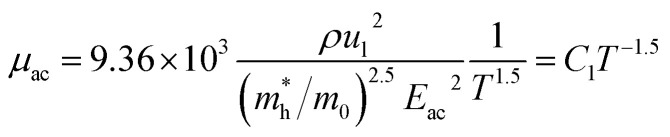
and13
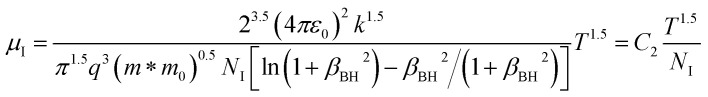
where14
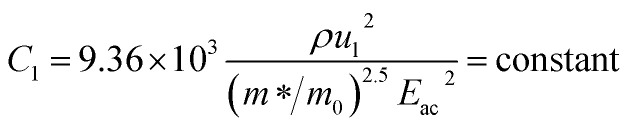
and15
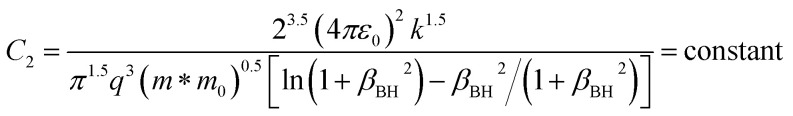


*ρ* is the density of β-FeSi_2_ (4.93 g cm^−3^), *u*_l_ is the sound velocity, *E*_ac_ = 0.5 eV is the deformation potential of acoustic phonons and *θ* = 640 K is the Debye temperature of the β-FeSi_2_ lattice.^53^ The sound velocity can be reviewed as follows:^55^16
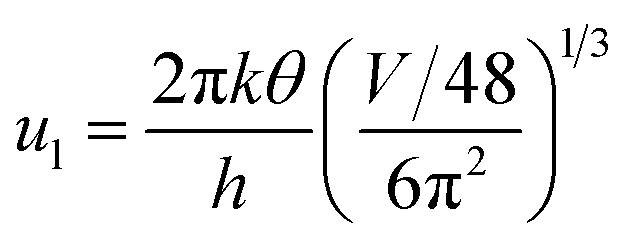


where *V* is the volume of the β-FeSi_2_ unit cell. The parameter *β*_BH_ is given by Brooks and Herring as^55^ follows:17
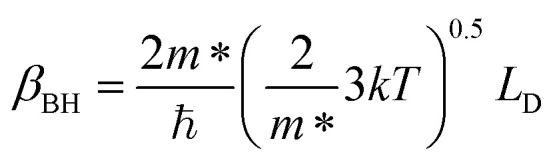
where *L*_D_ is the Debye length. Therefore, for two independent scattering mechanisms, *i.e.*, lattice and ionized impurity scatterings, the net mobility can be conveyed as a function of temperature by Mathiessen's rule as follows:^54^18
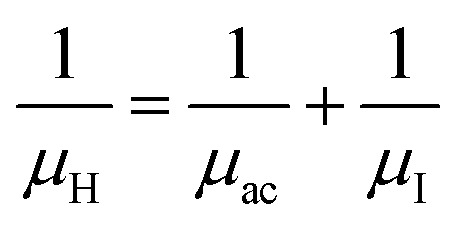


Hence, the expression for net mobility *μ*_ac_ and *μ*_I_ can be written as

19 1/*μ*_H_ = *AT*^1.5^ + *BN*_I_*T*^−1.5^

Henceforward, *A* and *B* coefficients have been determined by solving eqn (19) for several percentages (0.02%, 0.1% and 1%) of B dopant at room temperature. Therefore, the mobility (*μ*_H_) can be expressed as a function of temperature (*T*) for different dopings of B as follows:

20 *μ*_H_ = [3.4641 × 10^−5^*T*^1.5^ + 4.1859 × 10^−17^*N*_A_^−^*T*^−1.5^]^−1^ [for 0.02% B doping]

21 *μ*_H_ = [−8.1233 × 10^−5^*T*^1.5^ + 4.6413 × 10^−17^*N*_A_^−^*T*^−1.5^]^−1^ [for 0.1% B doping]

**Fig. 8:**
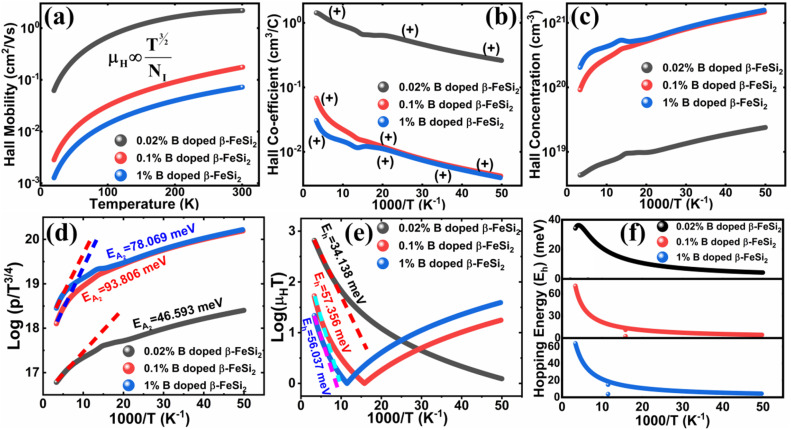
(a) The mobility of 0.02%, 0.1% and 1% B-doped β-FeSi_2_ as a direct function of temperature; (b) The Hall coefficient of 0.02%, 0.1% and 1% B-doped β-FeSi_2_ as a function of the reciprocal absolute temperature and (c) the Hall concentration of 0.02%, 0.1% and 1% B-doped β-FeSi_2_ as a function of reciprocal absolute temperature; (d) log(*p*/*T*^3/4^) of 0.02%, 0.1% and 1% B-doped β-FeSi_2_ as a function of the reciprocal absolute temperature; (e) log(*μ*_H_*T*) of 0.02%, 0.1% and 1% B-doped β-FeSi_2_ as a function of reciprocal temperature; (f) Hopping energies (*E*_h_) of 0.02%, 0.1% and 1% B-doped samples as a function of reciprocal temperature.

22 *μ*_H_ = [3.1215 × 10^−5^*T*^1.5^ + 4.4516 × 10^−17^*N*_A_^−^*T*^−1.5^]^−1^ [for 1% B doping]

where *N*_A_^−^ is the ionized acceptor concentration, which is equal to the hole (Hall) concentration at room temperature. Thus, the mobility (*μ*_H_) is plotted against temperature (*T*) for several B-dopings as presented in Fig. 8(a). Here, the mobility graph reveals only the rising segment of the mobility with temperature as *μ*_H_ ∝ *T*^3/2^, mostly due to ionized impurity scattering since the Hall carrier concentration is significantly higher owing to high defect densities in B-doped samples, which can be attributed to the interstitial doping of smaller B atoms in the β-FeSi_2_ lattice. With the increase in B doping from 0.02% to 1%, the mobility is quite significantly reduced from 2.2 to 0.07 cm^2^ V^−1^ s^−1^ as 
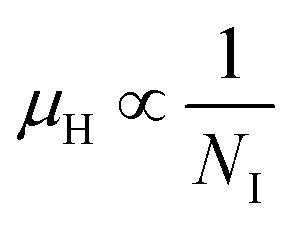
, which thereby signifies the occurrence of ionized impurity scattering. Thus, the trend of mobility variation with temperature and B doping validates the governance of ionized impurity scattering.

The temperature dependence of the Hall coefficient (*R*_H_) has been determined from the temperature dependence of mobility and resistivity using the following relation of 
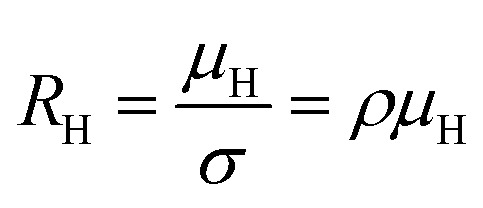
.^56^*R*_H_ has been plotted as a function of the reciprocal absolute temperature in Fig. 8(b). The sign of *R*_H_ is +ve over the entire temperature range for B-doped samples, indicating its conductivity to be p-type, which can be attributed to its majority carrier holes. The observed features of the temperature dependence of *R*_H_ for β-FeSi_2−2*x*_B_2*x*_ can be well justified by the dual-band model.^18–20^ As already stated, B-doped β-FeSi_2_ is considered to have a shallow defect band and a deep dopant level.^18–20^ At low and medium temperatures where *R*_H_ increases with temperature, it is considered to be affected by both kinds of holes from shallow and deep acceptor levels, whereas it is considered to be affected predominantly by holes based on deep acceptor levels at higher temperatures.^18,19^

Subsequently, the temperature dependence of the Hall concentration (*p*) was determined from the temperature dependence of *R*_H_ according to the following equation of 
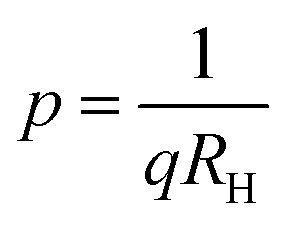
.^56^ Thus, Fig. 8(c) depicts the graph of the Hall concentration (*p*) as a function of reciprocal temperature (1000/*T*). Since resistivity is too low for B-doped samples (Table 4) and the Hall concentration is much higher due to dual acceptor levels, therefore, the region of increase in carrier concentration with temperature is absent in Fig. 8(c).^18,20^ The hole concentration does not increase much with the temperature in the semi-log scale, which can be attributed to the full ionization of the shallow acceptor band in the low-temperature regime.^52^ Nonetheless, the Hall concentration increases quite remarkably, almost in the order of two, with the increase in B doping from 0.02% to 1%. So far, the observed features of the Hall coefficient (*R*_H_) indicate the presence of dual-energy levels in doped β-FeSi_2_.^18,20^ Therefore, for B-doped β-FeSi_2_ containing a shallow acceptor band with energy *E*_A_1__ and a deep acceptor level with energy *E*_A_2__, the concentration of holes (*p*) can be reviewed from eqn (6).^20^ At high temperatures, disregarding the effects of the shallow defect band, the hole concentration (*p*) can be conveyed as already mentioned in eqn (9):^20^23
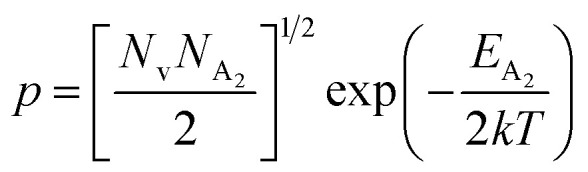
where24
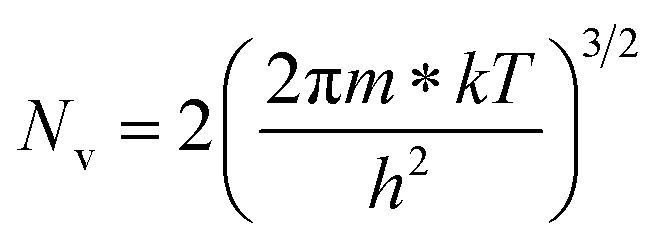


Therefore, putting the expression for *N*_v_ in eqn (23), the hole concentration can be revised as25
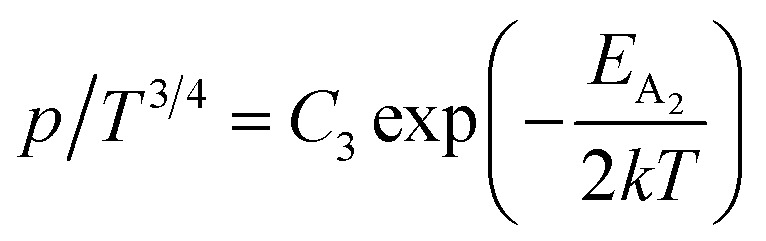
where26
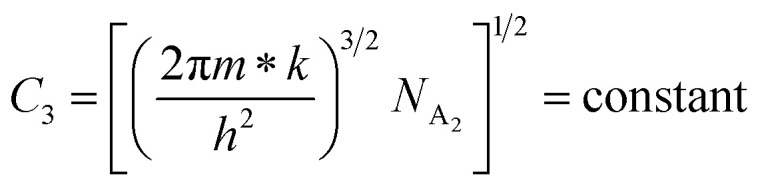


Taking logs on both sides, eqn (25) can be reviewed as follows:27
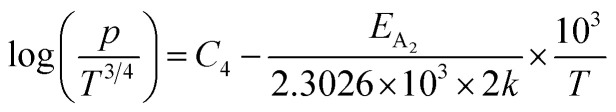
where 
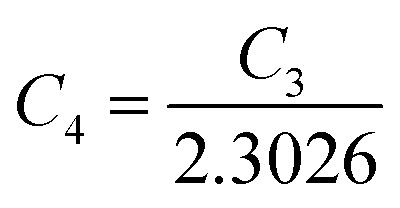
 is a constant.

Therefore, the plot of log(*p*/*T*^3/4^) as a function of inverse temperature (1/*T*) demonstrates a straight line at room temperature as well as high temperatures for various B dopings as shown in Fig. 8(d). Thus, the acceptor activation energy (*E*_A_2__) of doped β-FeSi_2_ at 300 K can be determined from the slope of those straight lines. *E*_A_2__ is estimated to be 46.593 meV, 93.806 meV and 78.069 meV for 0.02%, 0.1% and 1% B doping respectively. Therefore, the high-temperature activation energy of B-doped samples is reduced considerably from 93.806 meV to 78.069 meV as the doping increases from 0.1% to 1%, which agrees quite well with the literature.^18,19^

The S-like conductivity behaviour of Co and Mn-doped β-FeSi_2_ can be clarified by small polaron conduction within the temperature range 150–900 K, taking the crystalline distortion into account.^48^ Therefore, the electrical conduction of B-doped β-FeSi_2_ can also be justified by small polaron conduction as follows:^48,57^28
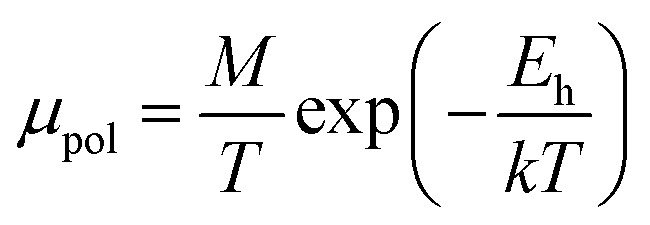
where *E*_h_ is the hopping energy and *M* is a constant factor independent of temperature. Hence, taking log on both sides, eqn (28) can be revised in linear form as follows:29
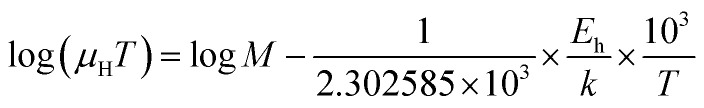


Thus, the logarithmic plot of (*μ*_H_*T*) in Fig. 8(e) as function of inverse temperature (10^3^/*T*) demonstrates linear behaviour whose slope 
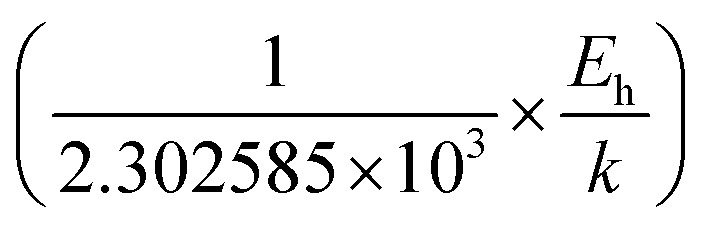
 at room temperature will lead to hopping energy (*E*_h_). With the increase in B doping from 0.02% to 1%, *E*_h_ increases significantly from 34.138 meV to 56.037 meV, which signifies that holes for lower B doping can be transported quite easily by lower thermal energy than those for higher B doping, which is in pretty good agreement with the results from Tani *et al.*^19^ Besides, *E*_h_ of the relatively lower (0.1%) B-doped sample is slightly higher (57.356 meV) than that (56.037 meV) of the highly (1%) B-doped sample, which may be accredited to the very nano nature of the 0.1% B-doped sample.

Hopping energy (*E*_h_) has been derived from the slope of the log(*μ*_H_*T*) *vs.* (10^3^/*T*) curve and plotted against the inverse of temperature (1/*T*) as revealed in Fig. 8(f). Typically, *E*_h_ increases with an increase in B doping from 0.02% to 1% specifically the peak hopping energy. Besides, the hopping energy peak shifts towards the higher temperature with an increase in B doping as can be perceived from Fig. 8(f).

##### 3.1.2.4 Analysis of Hall parameters from the dual-band model

As already stated, since Arushanov and Tani *et al.* both have proposed the existence of a dual band in Al and Mn-doped β-FeSi_2_, therefore, our B-doped sample can be estimated to have two energy levels: (i) a shallow acceptor band induced by defects, and (ii) a deep acceptor level due to B dopant.^18,20^ Thus, B-doped β-FeSi_2_ can be comprehended to have both kinds of holes, *i.e.*, (i) holes at the valence band with mobility *μ* and conductivity *σ*, and (ii) holes at the shallow acceptor band with mobility *μ*_1_ and conductivity *σ*_1_.^20^ Therefore, both kinds of holes contribute to the total electrical conductivity *σ*_0_ of the doped β-FeSi_2._ Hence, the hole concentration (*p*) for B-doped β-FeSi_2_ can be revised as cited earlier in eqn (6),^20^30
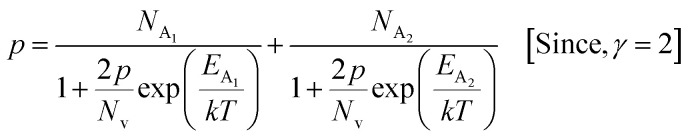


In the entire temperature range, considering 
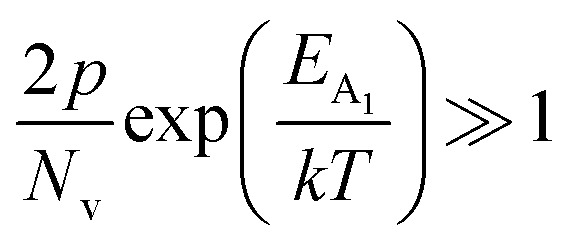
 and 
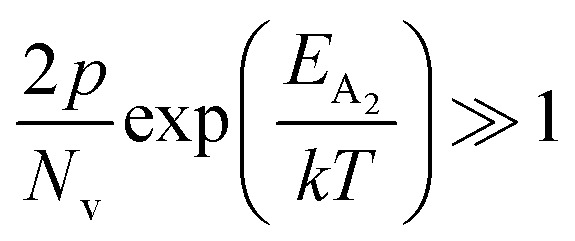
, the hole concentration (*p*) can be revised as follows:^20^31
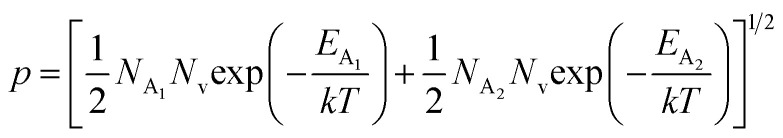


As already stated, the hole concentration (*p*) at low temperature can be expressed by just disregarding the effect of the deep acceptor level as mentioned in eqn (8):^20,52^32
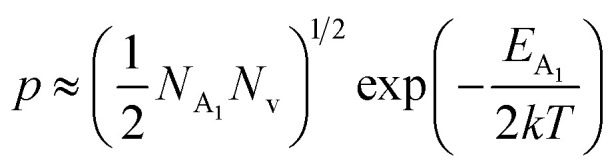


Therefore, taking log on both sides, eqn (32) can be revised as follows:33
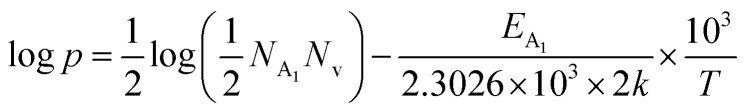


At high temperatures, the contribution due to the shallow acceptor band can be neglected quite easily and therefore, the hole concentration (*p*) can be revised as revealed earlier in eqn (9):^20,52^34
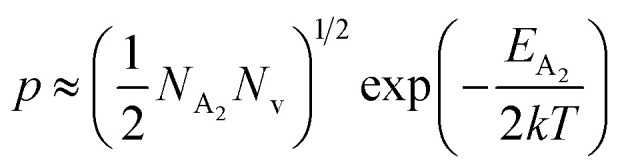


Thus, taking logs on both sides, eqn (34) can be revised as follows:35
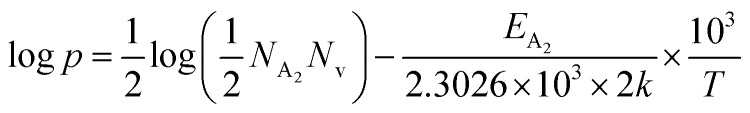


**Fig. 9:**
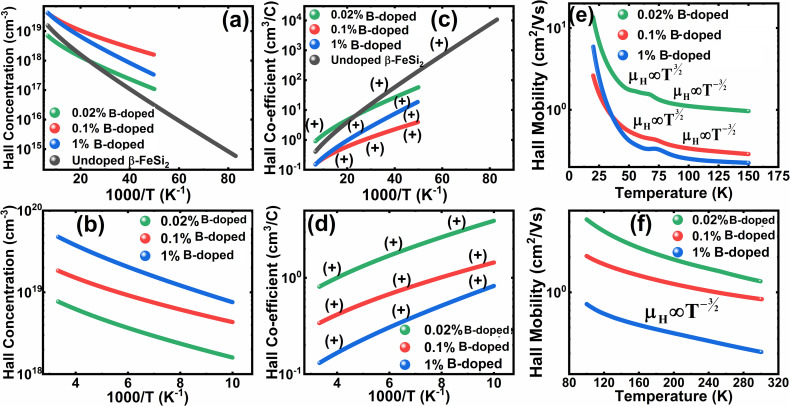
Hall concentration of undoped, 0.02%, 0.1% and 1% B-doped β-FeSi_2_ as a function of the reciprocal absolute temperature in the (a) low-temperature and (b) high-temperature regimes; The Hall co-efficient of undoped, 0.02%, 0.1% and 1% B-doped β-FeSi_2_ as a function of reciprocal absolute temperature in the (c) low-temperature and (d) high-temperature regime; The hopping energy (*E*_h_) of undoped, 0.02%, 0.1% and 1% B-doped β-FeSi_2_ as a function of the reciprocal absolute temperature in the (e) low-temperature and (f) high-temperature regime.

Thus, eqn (33) and (35) both portray linear relationships in between log *p* and the inverse of temperature (1/*T*) at low and high temperatures, respectively, as revealed in Fig. 9(a) and (b), which show that the Hall concentration (*p*) increased exponentially with an increase in temperature in both the low and high-temperature regimes. The Hall concentration (*p*) of the B-doped β-FeSi_2_ is significantly higher than that of the undoped one as can be observed from the low-temperature profile in Fig. 9(a). This can be attributed to the cumulative contribution of both deep as well as shallow acceptor levels to yield a larger number of carriers in doped β-FeSi_2_.^18,20,52^ At high temperatures, the trend is more or less identical to the low-temperature profile, *i.e.*, the carrier concentration becomes substantially higher with a gradual increase in B doping from 0.02% to 1%, which is essentially due to the full ionization of the deep acceptor level in the high-temperature regime.^18,52^ The hole concentration (*p*) of 0.1% B-doped β-FeSi_2_ is relatively higher than that of the 1% B-doped sample in the low-temperature regime, predominantly due to the substantial contribution of holes owing to higher defect densities in 0.1% B-doped nano-particles.

The temperature dependence of the Hall coefficient (*R*_H_) has been determined using the following equation: *R*_H_ = 1/*qp* and plotted against the reciprocal of temperature (10^3^/*T*) as shown in Fig. 9(c) and (d).^56^ Here, *R*_H_ is +ve for both undoped and B-doped β-FeSi_2_ in the low as well as high-temperature regions. Since *R*_H_ is the reciprocal of *n*_H_, it will exhibit just the converse property of the concentration (*p*) profile at both low and high temperatures, *i.e.*, log(*R*_H_) will increase almost linearly with a decrease in temperature. Moreover, as per the dual-band conduction Hall co-efficient, *R*_H_ can be reviewed as follows:^20^36
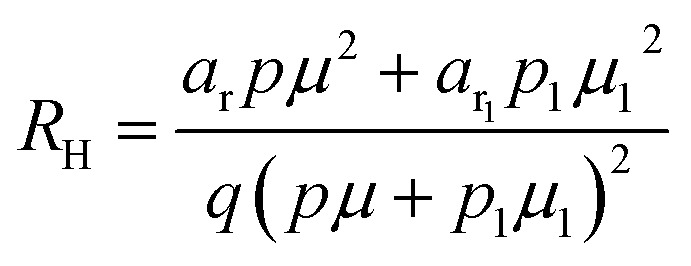
where *p* and *p*_1_ are hole concentrations in the valence band and the shallow acceptor band, respectively. Therefore eqn (36) signifies that *R*_H_ of the doped β-FeSi_2_ is considerably lower than that of the undoped one at both low and high temperatures. Thus, *R*_H_ is reduced remarkably with an increase in B doping for both the low and high-temperature regimes, essentially due to the full ionization of the deep acceptor level specifically in the high-temperature region.^18,52^ Nonetheless, the 0.1% B-doped sample exhibits *R*_H_ a bit lower than that of the 1% B-doped sample explicitly at low temperature. This was predominantly due to the substantial contribution of holes by the 0.1% B-doped β-FeSi_2_ owing to its higher defect densities caused by its doped nanoparticles.^48,52^

Subsequently, mobility (*μ*_H_) as a function of temperature at low as well as high temperatures was determined from the temperature dependence of *R*_H_ using the relation 
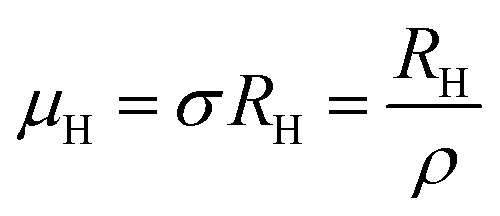
, as presented in Fig. 9(e) and (f).^56^ Essentially, the low-temperature profile of Hall mobility demonstrates a prominent peak between 70–75 K on both sides of which exhibits two projected sections: (i) a high-temperature segment above 75 K reveals the Hall mobility, which is inversely proportional to *T*^3/2^ (*μ*_L_ ∝ *T*^−3/2^) due to phonon scattering and (ii) the low-temperature segment below 75 K reveals Hall mobility, which is directly proportional to *T*^3/2^ and inversely proportional to the ionized impurity concentration (*N*_I_) 
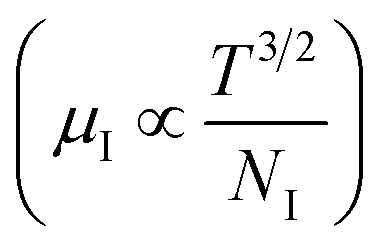
 owing to ionized impurity scattering. However, an effective low-temperature mobility profile as well as high-temperature profile validates the exponential decay of carrier mobility with temperature by following phonon scattering *μ*_H_ ∝ *T*^−3/2^. Specifically, the high-temperature trend dictates the gradual degradation of mobility with an increase in doping as can be perceived from Fig. 9(f). The carrier concentration (*p*) increases quite significantly with an increase in B doping due to the additional contribution of the deep acceptor level along with the shallow acceptor band leading to the substantial degradation of mobility.^18,20^

For B-doped β-FeSi_2,_ comprising of shallow and deep acceptor levels, the hole concentration (*p*) at high temperatures can be specified as already reviewed in eqn (27):^18,20,52^37
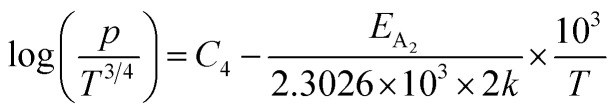


On the other hand, at low temperature, the hole concentration (*p*) can be written from eqn (32) as follows:^20,52^38
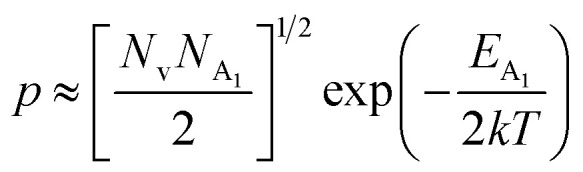


Henceforth, putting a value for *N*_v_ into eqn (38), the hole concentration (*p*) can be revised as follows:39
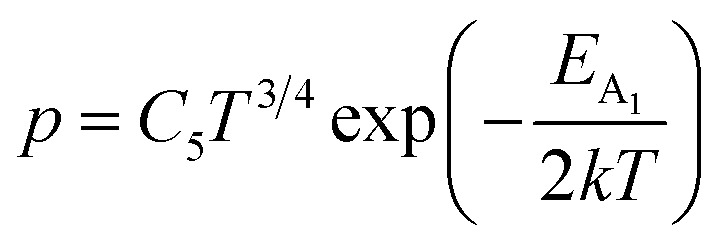


**Fig. 10:**
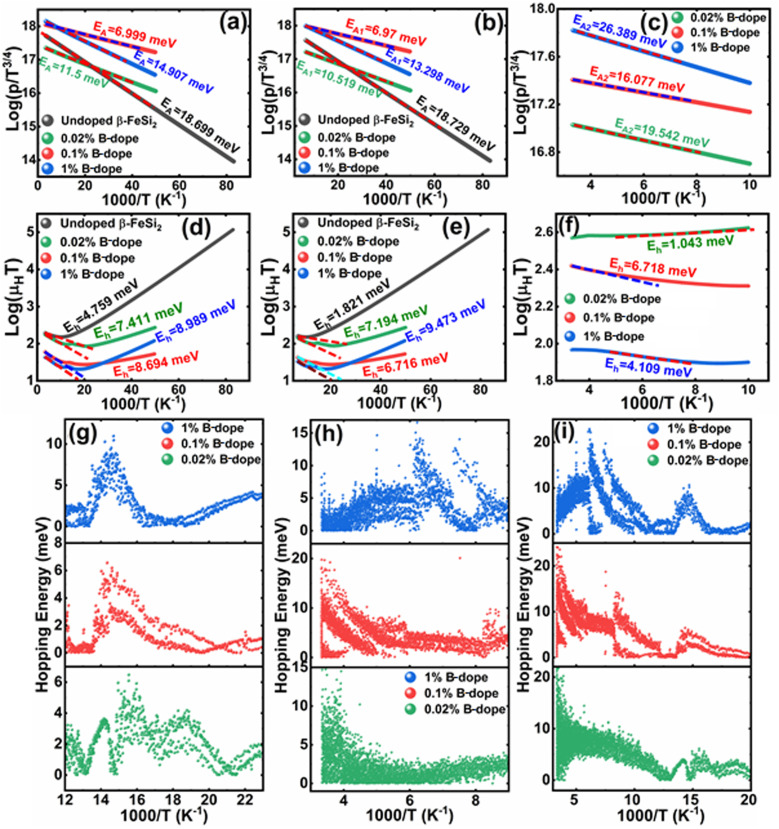
log(*p*/*T*^3/4^) of undoped, 0.02%, 0.1% and 1% B-doped β-FeSi_2_ as a function of the reciprocal absolute temperature in (a) the entire temperature regime of 20–300 K, (b) the low-temperature regime and (c) high-temperature regime; log(*μ*_H_*T*) of the undoped, 0.02%, 0.1% and 1% B-doped β-FeSi_2_ as a function of the reciprocal absolute temperature in the (d) entire temperature regime of 20–300 K, (e) low-temperature regime and (f) high-temperature regime; Hopping energy (*E*_h_) of 0.02%, 0.1% and 1% B-doped β-FeSi_2_ as a function of reciprocal absolute temperature in the (g) low-temperature regime, (h) high-temperature regime and (i) the entire temperature range of 20–300 K.

Taking log on both sides, eqn (39) can be redrafted as40
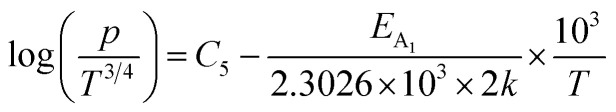
where *C*_4_ and 
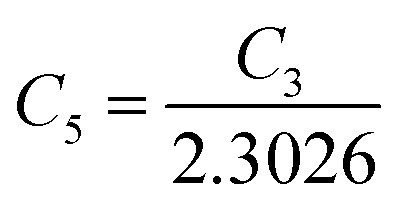
 are constants. Therefore, eqn (37) and (40) represent log(*p*/*T*^3/4^) as a linear function of the inverse temperature (1/*T*) at both high and low temperatures, respectively, as exhibited in Fig. 10(b) and (c), respectively. On the other hand, the linear relationship revealed in Fig. 10(a) is for the entire temperature range. Both shallow and deep acceptor activation energies *E*_A_1__ and *E*_A_2__ can be determined from the slopes of those straight lines at low and high temperatures, respectively. Besides, the activation energy (*E*_A_2__) of the deep acceptor level is considerably higher than that (*E*_A_1__) of the shallow acceptor band. *E*_A_1__ of 0.02%, 0.1% and 1% B-doped β-FeSi_2_ are 10.519, 6.97 and 13.298 meV, respectively, whereas the *E*_A_2__ of those doped samples are 19.542 meV, 16.077 meV and 26.389 meV respectively. Thus it indicates the clear existence of dual-energy levels in B-doped β-FeSi_2_.

In contrast, identical activation energies *E*_A_ (*E*_A_ ≈ 18.7 meV) in entire temperature ranges demonstrate the existence of only one acceptor level induced by defects in undoped β-FeSi_2_. Besides, the activation energy of the shallow acceptor band in doped β-FeSi_2_ is remarkably reduced compared to that of undoped β-FeSi_2_, which justifies the allocation of the shallow acceptor band substantially closer to the valence band in B-doped samples as compared to the undoped sample.

The mobility of B-doped β-FeSi_2_ can be conveyed as a function of temperature by following small polaron conduction within the entire temperature range of 20–300 K as follows, which is already cited in eqn (28):^48,57^41
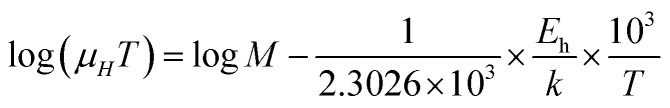


Thus, the slope of the linear reduction of log(*μ*_H_*T*) with the inverse of temperature (1/*T*) as per eqn (41) will provide the hopping energy (*E*_h_) of doped samples in the entire, high and low-temperature ranges as presented in Fig. 10(d)–(f) respectively. *E*_h_ increases significantly from 4.759 to 8.989 meV as the B doping increases from undoped to 1% doped conditions at room temperature as well as high temperatures, which agrees quite well with the report of Tani *et al.*^19^ Hopping energy (*E*_h_) also increases at low temperatures, from 1.821 to 9.473 meV, with an increase in B doping. Therefore, this fact strongly suggests that holes in low-doped β-FeSi_2_ can easily be transported by lower thermal energy than those for higher B doping.

Finally, the hopping energy (*E*_h_) has been determined from the derivative of the log(*μ*_H_*T*) curve and plotted as a function of inverse temperature (1/*T*) in Fig. 10(g)–(i) in the low, high and entire-temperature regimes, respectively. There is not much discrepancy in *E*_h_ (10–20 meV) for doped samples in the high-temperature regime, except from the shifting of the *E*_h_ peak towards lower temperature with an increase in B doping up to 1%. *E*_h_ becomes minimum, *i.e.*, very close to 1–2 meV at approximately less than 200 K explicitly for 0.02% and 0.1% B-doped β-FeSi_2_. The low-temperature profile in Fig. 10(g) demonstrates *E*_h_ maxima (∼5–10 meV) at approximately 66 K, whereas the hopping energy (*E*_h_) diminished to almost 1–2 eV after 80 K. The trend of hopping energy for the entire temperature range is more or less identical to that with the low-temperature profile within 50–100 K as exhibited in Fig. 10(i). Moreover, the value of the *E*_h_ peak increases and its range diminishes with an increase in B doping at low as well as high temperatures, including the entire temperature range.

### 3.2 Theoretical results

#### 3.2.1 Density of states and band structure analysis of pristine β-FeSi_2_

**Fig. 11:**
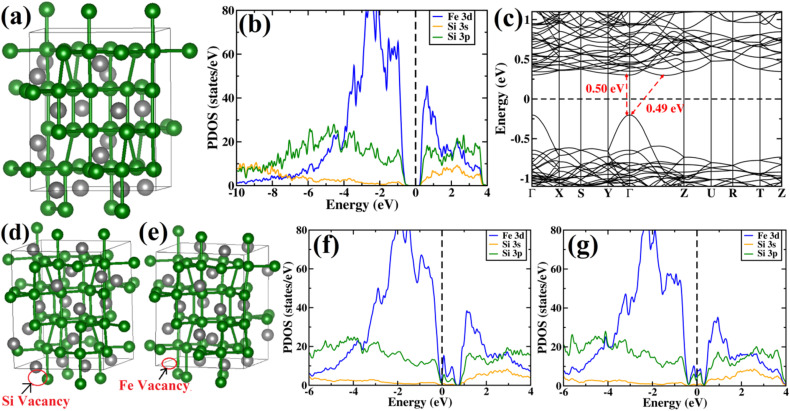
(a) The structure of the orthorhombic unit cell of β-FeSi_2_ consisting of 48 atoms. Color scheme for atomic spheres: Fe (gray) and Si (green); (b) projected density of states and (c) band structure of the orthorhombic cell of undoped β-FeSi_2_. The VBM and CBM are primarily contributed by Fe 3d and Si 3p states. There is an indirect gap of 0.49 eV between the *Γ* point and the point located at ⅔(*Γ*–*Z*); Structures of the orthorhombic unit cell of β-FeSi_2_ with a single (d) Si vacancy and (e) Fe vacancy in the unit cell. Color scheme for atomic spheres: Fe (gray) and Si (Green); Projected density of states of β-FeSi_2_ with (f) Si vacancy and (g) Fe vacancy. There is an additional peak occuring between the valence and conduction band due to the introduction of both Fe and Si vacancies.

We have performed *ab initio* density functional theory (DFT) calculations to realize the electronic structure of pristine β-FeSi_2_ and B-doped β-FeSi_2_. Initially, the structure of the orthorhombic unit cell of β-FeSi_2_ with 48 atoms was optimized using a variable cell relaxation technique. The optimized lattice parameters were 7.47 Å, 9.38 Å and 7.49 Å, respectively along the three crystallographic directions. The optimized structure of β-FeSi_2_ is shown in Fig. 11(a). Our values of optimized lattice parameters are in good agreement with the previously reported values calculated through DFT.^6,58^ There are four inequivalent atomic sites in the unit cell, two for Fe and two for Si denoted by Fe_I_, Fe_II_ and Si_I_, Si_II_, respectively. Primarily the projected density of states (PDOS) and band structure of pristine β-FeSi_2_ are shown in Fig. 11(b) and (c) respectively.

Subsequently, PDOS has been plotted essentially for the orbitals that have significant contributions in the valence band and conduction band near the band gap. From PDOS, it can be inferred that at low energies the valence band is comprised of mainly Si 3s and Si 3p orbitals. At higher energies near the valence band maximum (VBM), the contribution of Si 3p is more significant as compared to that of Si 3s. The majority of the contribution of valence band near the band gap is typically from Fe 3d states and there is a significant amount of hybridization between Fe 3d and Si 3p states. Also, there are a few Si 3s states near the VBM. Besides, at the conduction band, the major contribution is from the Fe 3d orbital similar to the valence band. Moreover, there is a significant amount of hybridization between Fe 3d, Si 3s and Si 3p states at the conduction band. From the band structure of β-FeSi_2_, an indirect gap of 0.49 eV can be easily observed between the *Γ* point and the point located at ⅔(*Γ*–*Z*). The value of the direct band gap at the *Γ* point is 0.50 eV, which is more than that of the indirect band gap. Therefore, our calculated band gap matches quite well with the previously reported theoretical results^58–60^ and is in good agreement with previous experimental results also.^1,2^

#### 3.2.2 Density of states analysis of undoped β-FeSi_2_ with Si and Fe vacancies

There have been very few studies that support the dual-band model on β-FeSi_2_.^18–20^ Also, our experimental results strongly suggest the dual-band model for B-doped β-FeSi_2_. According to the model,^18–20^ the doped system has two acceptor levels: (i) a shallow acceptor level due to structural defects and (ii) a deep acceptor level due to dopant. There have been studies demonstrating that the most common structural defect in β-FeSi_2_ mainly comes from Fe and Si vacancies.^61,62^ The optimized structures for the unit cell of β-FeSi_2_ with Si vacancy and Fe vacancy are exhibited in Fig. 11(d) and (e), respectively. The PDOS of β-FeSi_2_ with Si and Fe vacancies are shown in Fig. 11(f) and (g), respectively, as compared to the pristine structure (compare Fig. 11(b) with Fig. 11(f) and (g)). Thus, there is an additional peak rise between the valence band and conduction band as compared to the pristine structure, basically due to the introduction of vacancies. Since the pristine structure is non-magnetic, therefore, magnetic calculations were also performed to determine whether the vacancy structure was magnetic or not. However, it has been found that for both Fe and Si vacancies, the system is non-magnetic. From Fig. 11(f), it can also be inferred that the vacancy peak mainly comes from the hybridization of Fe 3d and Si 3p states; also, the Fermi energy lies at the top of the valence band. Hence, it elucidates the p-type conductivity of the β-FeSi_2_ lattice system having Si vacancy. Besides, Fig. 11(g) also demonstrates that the vacancy peak with Fe vacancy has the same hybridization as that with Si vacancy; however, here, the Fermi level lies at the middle of the peak, which signifies the n-type conductivity of β-FeSi_2_ with Fe vacancy. However, from experiment, it has been investigated that the structural defects of our β-FeSi_2_ sample are mainly acceptor type, which designates that the Si vacancies are more abundant in our structure than that of Fe vacancies.

#### 3.2.3 Density of states analysis of boron-doped β-FeSi_2_

**Fig. 12:**
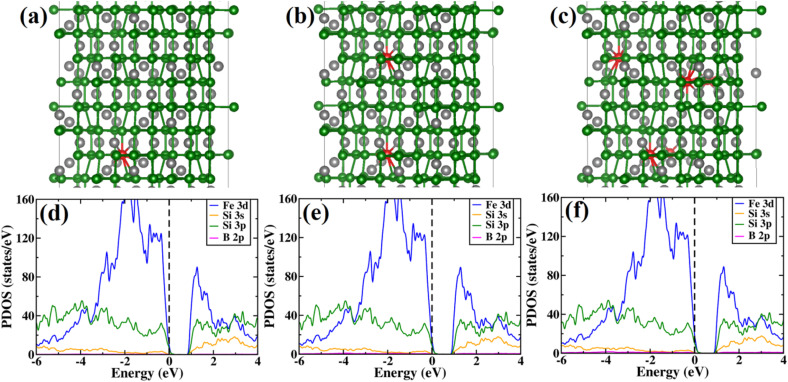
Structures of B-doped β-FeSi_2_ for (a) 0.78% B doping, (b) 1.56% B doping and (c) 2.34% B doping. Color scheme for atomic spheres: Fe (gray), Si (green) and B (red); Projected density of states for β-FeSi_2_ for (d) 0.78% B-doped β-FeSi_2_, (e) 1.56% B doped β-FeSi_2_ and (f) 2.34% B doped β-FeSi_2_.

The variations of the band gap and atomic levels with B doping were also considered. To obtain close proximity to experimental B doping concentration, we have considered the 2 × 2 × 1 supercell of β-FeSi_2_. Thus, Si was substituted by B, corresponding to 0.78%, 1.56% and 2.34% of B doping. The optimized structure for the three doping percentages of B are shown in Fig. 12(a)–(c). In Fig. 12(d)–(f), the PDOS of β-FeSi_2_ has been demonstrated for various concentrations of B doping. Although the structure of pristine β-FeSi_2_ is non-magnetic, it has been found that upon inclusion of B dopant, the system remains non-magnetic as well. Moreover, from the projected density of states in Fig. 12(a)–(c) it has been observed that upon B doping, the Fermi energy lies at the top of the valence band, thus illustrating the p-type conductivity of B-doped β-FeSi_2_.

#### 3.2.4 Charge density analysis of boron-doped β-FeSi_2_

**Fig. 13:**
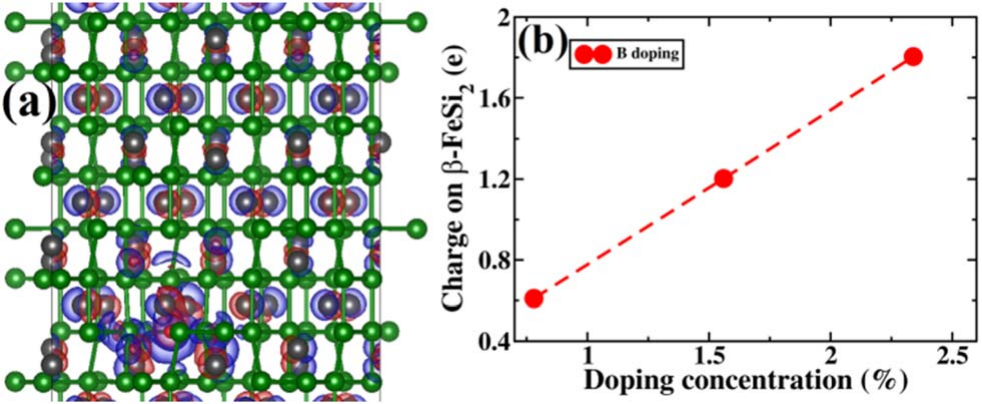
(a) The charge density difference between B-doped and pristine β-FeSi_2_. Red and green lobes correspond to the gain and loss of electronic charge, respectively. The iso-surface corresponding to 0.005 e (bohr)^−3^ has been plotted; (b) The charge on β-FeSi_2_ as a function of B doping concentration has been plotted.

Successively we have tried to visualize the charge density due to doping. Since only electronic density has been obtained from our calculations, it is very difficult to realize the effect of p-type doping due to B inclusion. Therefore, the charge density difference has been taken between the doped system and pristine β-FeSi_2_. In Fig. 13(a), we have shown the charge density difference between the B-doped sample and pristine β-FeSi_2_. Red and green lobes correspond to the gain and loss of electronic charge, respectively. The iso-surface corresponding to 0.005 e (bohr)^−1^ has also been plotted. Therefore, it can be inferred that the B-doped system has p-type behavior from the difference in densities of the red lobe and blue lobe near the dopant B.

Next, the amount of charge transfer to β-FeSi_2_ due to B doping was quantified. Here, the procedure as suggested by Bader *et al.* was followed, which makes use of the topological properties of the charge density.^63^ Sign convention has been used so that if the charge is negative, electrons are supposed to transfer to β-FeSi_2_, making the material n-type, whereas if the charge is positive, then electrons are supposed to transfer from β-FeSi_2_, making the material p-type. Thus, from Fig. 13(b), it can be inferred that as the system is doped with B, the electrons are transferred from the β-FeSi_2_ system to the dopant, making it a p-type material and with further increase in B doping, the number of holes increases linearly, increasing the p-type nature of the system. Thus the consequence matches quite well with our experimental results. Although the experimental and theoretical doping concentrations are a bit different, the general trends remain the same.

In summary, we have clarified the dual-band model considering the β-FeSi_2_ lattice structure with vacancy defects and B doping. It has been exhibited that the Si vacancy in β-FeSi_2_ makes the system p-type, whereas Fe vacancy in β-FeSi_2_ makes the system n-type. Therefore depending on the synthesis procedure each type of vacancy can vary and the system behaves accordingly; *i.e.*, from our experiment, it was found that the structural defect was mainly the acceptor type, and thus the amount of Si vacancy was substantially higher as compared to that of Fe vacancy in our system. Thereafter, it has been shown how B doping modulates the system to p-type material and how the amount of charges changes with B doping. We have also computed the amount of charge transfer to the system upon B doping, which shows the theoretical results to be in excellent agreement with the experimental one.

## 4. Conclusions

β-FeSi_2_ was doped with B *via* a simple and cost-effective chemical reduction technique, using ortho-boric acid as a precursor while synthesizing iron di-silicide. In B doping, smaller boron atoms substitute larger Si_II_ atoms, which lead to the reduction in the inter-planar spacing, *d*, to further result in the right shifting of XRD peaks towards higher diffraction angle. On the other hand, blue shifting and broadening of β-Raman peaks are also observed as a result of doping, due to the decrease in bond length. Moreover, the extensive up-shift in the binding energy of the Si 2p, Fe 2p and Fe 3p peaks confirms the reality of B doping. Besides, the weak XPS peak of B 1s also infers the presence of B dopants.

The electrical properties of B-doped β-FeSi_2−2*x*_B_2*x*_ were investigated within the temperature range of 20–300 K. The Hall investigation demonstrates that B-doped β-FeSi_2_ is a p-type semiconductor. Besides, with an increase in B doping from 0.02% to 1%, the resistivity (*ρ*) decreases from 0.66–0.4 Ω cm at room temperature. The Hall parameters were also analyzed as a function of temperature using thermal mobility and the dual-band model to explore their properties. The trend of mobility increament with temperature following *μ*_H_ ∝ *T*^3/2^ as well as the trend of mobility reduction with B doping following 
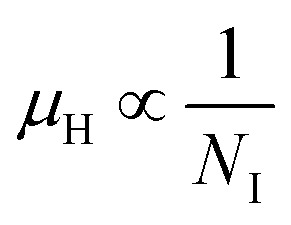
 signify the dominance of ionized impurity scattering. The temperature profile of *R*_H_ demonstrates the contribution of both kinds of holes from shallow and deep acceptor levels at low temperatures, whereas it is predominantly affected by holes from deep acceptor levels at high temperatures.

The dual-band model investigation reveals that the Hall concentration (*p*) increases exponentially with an increase in temperature and also becomes substantially higher with a gradual increase in B doping due to the cumulative contribution of deep and shallow acceptor levels in the entire temperature regime. It can also be attributed to the full ionization of deep acceptor levels in the high-temperature regime. The low-temperature mobility profile demonstrates both sections of phonon and ionized impurity scattering just above and below 75 K, respectively. Besides, the high-temperature activation energies of B-doped samples are considerably higher than those of low-temperature energies, which demonstrates the clear evidence of the dual-band model. Moreover, the hopping energy trend from the dual-band analysis demonstrates the fact that holes in low-doped samples can be transported quite easily by lower thermal energy as compared to higher B doping, which is in good agreement with the mobility model analysis. Therefore, the above electrical characterizations precisely investigates and validates the effective B doping of β-FeSi_2_.

Besides, from density functional theory calculations, we have demonstrated the origin of the dual-band model from the electronic structure of β-FeSi_2_. It has also been presented how the Si and Fe vacancies make the system p-type and n-type, respectively. Furthermore, the effect of B doping on the electronic structure of β-FeSi_2_ has also been demonstrated. The charge transfer to the system has been computed due to B doping, which signifies the system to be more of a p-type with an increase in B doping. Thus, the theoretical results are in excellent agreement with the experimental outcomes. Therefore, this β-phase of iron silicide doped with a simple chemical technique will be very useful for fabricating efficient p–n homo and hetero-junction-based efficient solar cells.

## Conflicts of interest

The authors declare that the research manuscript has not been published in part or in entirety, nor under consideration by other journals, no competing conflict is involved in this manuscript.

## Supplementary Material
